# High Affinity Antibodies against Influenza Characterize the Plasmablast Response in SLE Patients After Vaccination

**DOI:** 10.1371/journal.pone.0125618

**Published:** 2015-05-07

**Authors:** Kaval Kaur, Nai-Ying Zheng, Kenneth Smith, Min Huang, Lie Li, Noel T. Pauli, Carole J. Henry Dunand, Jane-Hwei Lee, Michael Morrissey, Yixuan Wu, Michelle L. Joachims, Melissa E. Munroe, Denise Lau, Xinyan Qu, Florian Krammer, Jens Wrammert, Peter Palese, Rafi Ahmed, Judith A. James, Patrick C. Wilson

**Affiliations:** 1 Committee on Immunology, The University of Chicago, Chicago, Illinois, United States of America; 2 Department of Medicine, Section of Rheumatology, The Knapp Center for Lupus and Immunology, The University of Chicago, Chicago, Illinois, United States of America; 3 Arthritis and Clinical Immunology Program, Oklahoma Medical Research Foundation, Oklahoma City, Oklahoma, United States of America; 4 Immunobiology and Cancer Research Program, Oklahoma Medical Research Foundation, Oklahoma City, Oklahoma, United States of America; 5 Department of Microbiology, Icahn School of Medicine at Mount Sinai, New York, New York, United States of America; 6 Emory Vaccine Center, Department of Microbiology and Immunology, Emory University School of Medicine, Atlanta, Georgia, United States of America; 7 Department of Medicine and Pathology, University of Oklahoma Health Sciences Center, Oklahoma City, Oklahoma, United States of America; Pavillon Kirmisson, FRANCE

## Abstract

Breakdown of B cell tolerance is a cardinal feature of systemic lupus erythematosus (SLE). Increased numbers of autoreactive mature naïve B cells have been described in SLE patients and autoantibodies have been shown to arise from autoreactive and non-autoreactive precursors. How these defects, in the regulation of B cell tolerance and selection, influence germinal center (GC) reactions that are directed towards foreign antigens has yet to be investigated. Here, we examined the characteristics of post-GC foreign antigen-specific B cells from SLE patients and healthy controls by analyzing monoclonal antibodies generated from plasmablasts induced specifically by influenza vaccination. We report that many of the SLE patients had anti-influenza antibodies with higher binding affinity and neutralization capacity than those from controls. Although overall frequencies of autoreactivity in the influenza-specific plasmablasts were similar for SLE patients and controls, the variable gene repertoire of influenza-specific plasmablasts from SLE patients was altered, with increased usage of JH6 and long heavy chain CDR3 segments. We found that high affinity anti-influenza antibodies generally characterize the plasmablast responses of SLE patients with low levels of autoreactivity; however, certain exceptions were noted. The high-avidity antibody responses in SLE patients may also be correlated with cytokines that are abnormally expressed in lupus. These findings provide insights into the effects of dysregulated immunity on the quality of antibody responses following influenza vaccination and further our understanding of the underlying abnormalities of lupus.

## Introduction

During normal B cell development, most autoreactive B cells are removed by tolerance mechanisms. This is evident in the drastic decrease in the numbers of self-reactive B cells in healthy individuals from 76% of the early immature compartment to 20% of the mature naïve B cell compartment [1]. However, in patients with systemic lupus erythematosus (SLE), primary B cell tolerance fails and there is instead an accumulation of self-reactive mature naïve B cells (44%), which persists even in patients who are in clinical remission [2, 3].

It was recently demonstrated in a mouse model that autoreactive B cells that recognize both self and foreign antigens can be recruited into germinal centers (GCs), where somatic hypermutation can reduce reactivity to self but maintain reactivity to the foreign antigen [4]. In the context of SLE, it is unclear whether autoreactive B cells participate in immune responses to foreign antigens. Autoreactive precursors do give rise to autoantibodies, which are the hallmark phenotype of SLE [5]. However, so do non-autoreactive precursors by somatic hypermutation and affinity maturation against self-antigens, indicating that maintenance of secondary tolerance in the germinal centers (GCs) of SLE patients is also defective [5–9]. Autoreactive 9G4 B cells encoded by VH4-34, that fail to progress past the early stages of GC reactions in healthy individuals, are significantly expanded in the post-GC IgG memory and plasma cell compartments of SLE patients [10, 11]. Hence, the participation of autoreactive B cells in immune responses against foreign antigens could potentially lead to increased autoreactive responses in SLE patients. Whether pathological autoantibodies arise in SLE patients during foreign-antigen immune responses is an important concern in the context of infections and vaccinations. Reports of plasmablast frequencies in SLE patients that wane and swell relative to disease state suggest that there are ongoing or recurrent autoimmune responses induced by either self- or foreign antigens [12, 13]. However, on the whole, SLE patients and healthy controls have been reported to have similar overall frequencies of IgG memory B cells that are autoreactive [5]. With regards to vaccinations, several studies have shown that at the serological level, SLE disease activity is generally not altered after vaccination, although in certain patients temporary increases in serum autoantibody titers may be observed [14–18]. It is not known if these increases in autoantibody titers signify increased self-antigen immune responses or increased levels of cross-reactivity to self-antigens in the foreign antigen-specific compartment following vaccination.

The impact of both an autoreactive B cell repertoire and defective secondary tolerance on the quality of immune responses to foreign antigens is not well characterized. It is unclear how the binding characteristics of foreign antigen-specific B cells are altered in SLE patients, who have several other immune system abnormalities. Thus far, antibody responses of SLE patients to vaccinations have been studied only at the level of serology. Distinguishing between the quality of individual antigen-specific B cells from the overall quantity of the polyclonal antibody response is challenging when relying only on serum. Furthermore, some studies report no significant differences in the serum antibody titers between vaccinated SLE patients and controls [19, 20] while others report that SLE patients have lower serological responses [14, 21, 22]. This may be a consequence of the heterogeneity of the patient cohorts and factors such as lymphopenia, which influence the quantity of the response [17, 18, 23, 24]. It has been reported that patients experiencing disease flares or having higher titers of antinuclear antibodies generally have lower responses to vaccination [17]. This could indicate that the participation of autoreactive B cells, naïve or memory, in GC reactions against foreign antigens may be detrimental to the quality of the response.

Alternatively, the participation of autoreactive B cells in foreign-antigen immune responses may be advantageous. Broadly neutralizing HIV antibodies (BnAbs) isolated from infected individuals are autoreactive and bind strongly to autoantigens [25–27]. It has been suggested that SLE patients might be able to develop these BnAbs more efficiently as they are unable to delete autoreactive B cells [28–30]. This may explain the disproportionately low occurrence of HIV infections in SLE patients [31–33]. It has also been reported that polyreactivity increases the avidity of polyreactive anti-HIV antibodies via heteroligation between a high affinity HIV epitope and a low affinity polyreactive site [27]. Having an autoimmune B cell repertoire that escaped tolerance could also mean that there is a broader repertoire and wider spectrum of specificities available to participate in immune responses, thus improving protective responses against pathogens. The mature naïve B cell repertoire of SLE patients exhibits abnormalities in the immunoglobulin (Ig) gene usage, with biases toward VH3, VH4-34, VK1 and VK4 gene families [1, 3, 11, 12, 34]. How this altered repertoire affects the make-up of foreign antigen-specific B cells could provide insights into the changes in B cell immunopoeisis in SLE patients.

Here, we investigated the B cell antibody responses in a cohort of SLE patients to characterize their post-GC foreign antigen-specific compartment in terms of antigen binding capacities and level of autoreactivity. We generated monoclonal antibodies from acutely activated B cells, plasmablasts, isolated from SLE patients and control subjects 7 days after influenza vaccination. We report that influenza-positive antibodies from the SLE patients had higher avidity and neutralization capacities against influenza than those from controls. Similar overall frequencies of autoreactivity in the influenza-positive antibodies were seen in both cohorts, though differences in HEp-2 reactivity were noted. We describe distinct features in the gene repertoire of influenza-positive antibodies from SLE patients, and provide data suggesting that the enhanced immune responses seen in SLE patients may be a consequence of cytokines that are abnormally expressed in lupus. This study shows that at the level of individual plasmablasts, some SLE patients can generate a higher affinity response against foreign antigens than controls. This altered response may reflect the underlying abnormalities due to lupus and investigating the causes of the altered B cell responses could provide new avenues for delineating the cause of lupus.

## Materials and Methods

### Patients and control subjects

The Oklahoma Medical Research Foundation and the University of Chicago institutional review boards approved this study. Each volunteer provided his or her written informed consent. Detailed information on the 10 SLE patients and 8 controls recruited for this study can be found in S1 Table. Subjects that received vaccination in the 2006/07 vaccinating season were S2, S6, S8, S10, C1, C3, C5, C6 and C8. Subjects that received vaccination in the 2007/08 season were S1-8, S10 and C1-8. Subjects that received vaccination in the 2008/09 season were S1-6, S8, S9, C1-4 and C6-8.

### Monoclonal Antibodies

Antibodies were generated as previously described [35]. Briefly, peripheral blood was obtained from each individual 7 days after influenza vaccination was administered. Lymphocytes were purified and enriched for B cells with treatment of RosetteSep. Plasmablasts (CD3^-^CD19^+^CD20^low^CD27^hi^ CD38^hi^) were single-sorted into 96-well plates, and RT-PCR, followed by nested PCR, were used to amplify the variable heavy and light chain antibody genes on a single-cell basis. The sequences were then cloned into IgG expression vectors and transfected into HEK293 cells. HEK293 cells were obtained from American Type Culture Collection (ATCC). Five days after transfection, antibodies were purified from the culture supernatant using protein A agarose beads (Thermo Scientific) and subsequently concentrated.

### Virus and rHA ELISA

Influenza viruses were freshly grown in eggs, and purified as previously described [36]. 8 hemagglutination units (HAU) of virus were used for ELISA. Monoclonal antibodies were serially diluted 1:3 starting at 30ug/ml and tested for reactivity against the three virus strains (H1N1, H3N2 and B) in the corresponding vaccine and 1ug/ml of the respective rHA proteins (BEI Resources, NIAID, NIH) by ELISA. Goat anti-human IgG peroxidase conjugate (Jackson ImmunoResearch) was used to detect antibody binding. ELISA results for the virus strain each antibody was most specific against, was used for analysis. K_D_ values of antibody binding were determined by Scatchard analysis using nonlinear regression (one site binding model) on GraphPad Prism software.

### Surface plasmon resonance

Kinetic interactions of the monoclonal antibodies (IgGs) with rHA protein were measured on the ProteOn XPR36 (BioRad). Experiments were performed in HBS-EP running buffer. 20ug/ml of goat anti-human IgG Fc fragment specific antibody (Jackson ImmunoResearch) in acetate buffer pH5.5 was immobilized on a GLC chip at 30ul/min by amine coupling resulting in around 3000RU. 5ug/ml of each antibody of interest was injected in running buffer at a flow rate of 40ul/min for 60s for capture by the immobilized anti-IgG. For kinetic measurements, rHA at five different concentrations ranging from 0.78125nM to 50nM in HBS-EP buffer was injected at flow rate of 25ul/ml with 5 min association and 4 hour dissociation times. The surface was regenerated by two injections of 0.85% phosphoric acid at 100ul/min for 18s each. This removed antibody that was captured by the anti-IgG; hence leaving the immobilized anti-IgG available for capturing another round of antibodies. For data analysis, double subtraction (interspot (blank reference) and running buffer) was done. Affinities, or K_D_ values, were calculated by aligning the data to 1:1 binding two-state model using ProteOn Manager v.3.1 (BioRad).

### Hemagglutination inhibition assay

Influenza-positive monoclonal antibodies were tested for ability to inhibit hemagglutination of the virus strain they bound. 8HAUs of virus in 25ul of PBS were incubated with 25ul of two-fold diluted antibodies (60–0.234ug/ml) in duplicate for 30 minutes. 50ul of 1% of PBS-washed Turkey RBCs (Lampire Biological Laboratories) was then added. The minimum effective concentration of each antibody was the lowest concentration of antibody that inhibited viral hemagglutination of RBCs.

### Microneutralization assay

Influenza-positive monoclonal antibodies were tested for neutralization capacity against the virus strain they bound as previously described [37]. 100TCID_50_ of virus in 50ul DMEM was incubated with 50ul of three-fold diluted antibodies (60–0.027ug/ml) in triplicate at 37^°^C for 1 hour. This mixture was then added to PBS-washed MDCK cells for another hour. MDCK cells were obtained from American Type Culture Collection (ATCC). Cells were then washed and incubated in DMEM supplemented with 100 U/ml penicillin and 100 mg/ml streptomycin, 0.5% BSA and 0.5 μg/ml TPCK-Trypsin for 70 hours. The minimum effective concentration of each neutralizing antibody was the lowest concentration of antibody that inhibited viral infection as identified by the HA assay.

### Polyreactivity, HEp-2 ELISAs and immunofluorescence assay (IFA)

All antibodies were tested for polyreactivity and HEp-2 cell reactivity (INOVA Diagnostics) by ELISA as previously described [1]. Antibodies were considered polyreactive when they bound at least two of the following three antigens with an OD greater than 0.5: dsDNA, insulin and lipopolysaccharide. For HEp-2 IFA, 50ug/ml of antibody was incubated on HEp-2 coated slides (MBL Bion) for 30 min, washed with PBS and binding detected using FITC-conjugated anti-human antibody by fluorescence microscopy. Positive and negative control sera used were included in the kit. HEp-2 IFA reactivity was scored on a scale of 0–4, with 4 being the highest level of reactivity, in a blinded manner by ten volunteers. The mode of the scores was used for analysis.

### Sequence analysis

Variable heavy and light chain antibody genes were analyzed for gene usage, mutations, CDR3 length and CDR3 isoelectric point (pI) using *JOINSOLVER* and the IMGT database.

### Antibody Microarray

Antibodies were diluted in protein printing buffer to 250ug/ml and printed on SuperEpoxy glass slides using the SpotBot 3 microarrayer (ArrayIt). Antibodies were spotted in triplicate on each array. Printed slides were stored overnight at 4°C in the dark. Before each use, slides were washed three times with PBS 0.05% Tween-20 and then treated with BlockIt blocking buffer (ArrayIt) for 1 hr at room temperature. Slides were washed three times with protein microarray wash buffer in between each step and all the reactions were done in protein microarray reaction buffer (ArrayIt). To detect the antibodies spotted and normalize the signals, the slides were then incubated for 1 hr with Cy3-conjugated goat anti-human IgG, Fc-fragment specific (Jackson). After washing, the slides were fitted with a 24 well hybridization cassette that allowed for several different biotinylated rHAs at 20ug/ml each to be applied to a single array for 1 hr. rHAs were biotinylated the previous day with EZ-Link Maleimide-PEG2-Biotin (ThermoScientific). To detect bound rHA, slides were incubated with Alexa-Fluor 647-conjugated Streptavidin (Invitrogen) for 1 hr. Signals were quantified by a GenePix 4000B microarray scanner and analyzed with GenePix Pro 6.0 software (Molecular Devices).

### Multiplex bead assay

Day 0 serum samples from 7 SLE patients and 7 control individuals were tested in duplicate for 32 cytokines using xMAP multiplex bead-based assay (Affymetrix) or ELISA (BLyS (R&D systems) and APRIL (eBiosciences)).

### Statistical analysis

P values were calculated by comparing means using unpaired t-test or medians using Mann-Whitney test with significance <0.05. All statistics were done with two-tailed analyses using GraphPad Prism software. Spearman’s nonparametric correlation test with two-tailed analysis was used to test the correlation between K_D_ values measured by virus ELISA and SPR.

## Results

### Generation of influenza-specific monoclonal antibodies from SLE patients after influenza vaccination

To investigate the quality of antibody responses to foreign antigens in SLE patients, we expressed monoclonal antibodies (mAbs) from the cloned variable genes of single plasmablasts isolated from the 10 SLE patients and 8 healthy controls 7 days after they had received the trivalent seasonal influenza vaccine (S1 Table). Plasmablasts are activated B cells found in the peripheral blood only transiently after vaccination or infection and are typically antigen-specific [35, 36]; therefore, in contrast to serum antibodies or memory B cells, plasmablasts represent the B cells specifically activated by ongoing immune responses only. While the percent of total B cells was lower in the SLE patients compared to controls, the percent of plasmablasts (CD3^-^CD19^+^CD20^low^CD27^hi^ CD38^hi^) out of total B cells was similar. In total, 446 mAbs were generated, out of which 131 antibodies from SLE patients and 124 antibodies from controls bound to influenza virus, as detected by enzyme-linked immunosorbent assay (ELISA) (S2 Table). Antibodies from each subject were tested against each of the three virus strains (H1N1, H3N2 and B) present in the trivalent seasonal influenza vaccine. This allowed us to identify the virus strain specificity of each antibody and the following results were obtained by testing each antibody with its corresponding virus strain. Similar proportions of influenza-positive antibodies from SLE patients and controls bound to each of the three different virus subtypes.

### Influenza-positive antibodies from most SLE patients have higher avidities

**Fig 1:**
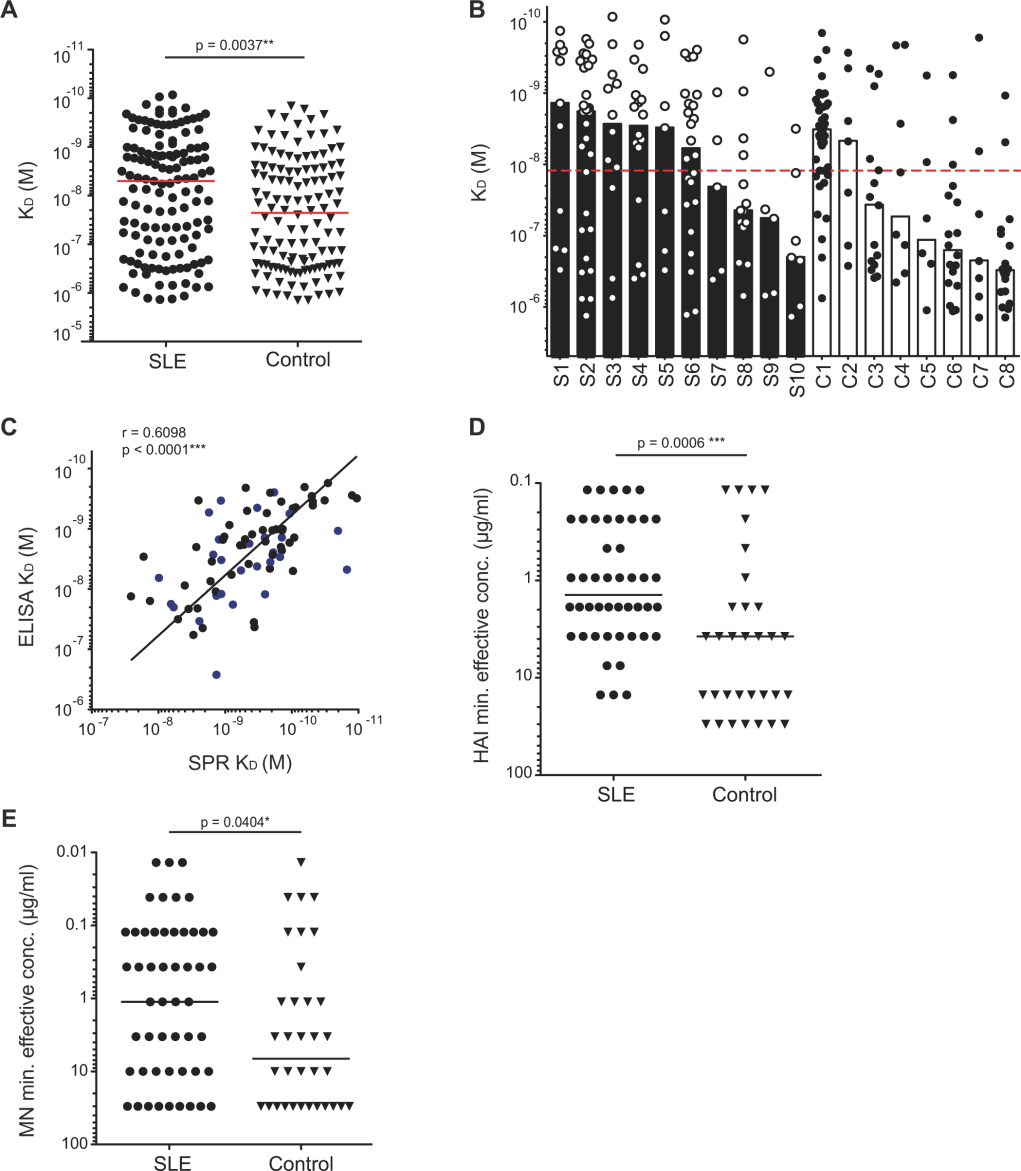
Higher avidities and neutralization capacities observed for monoclonal antibodies from SLE patients. (A) Monoclonal antibodies generated from plasmablasts isolated from subjects were tested for virus binding by ELISA. Binding avidities (K_D_) were estimated by Scatchard plot analyses of ELISA data. K_D_ values of pooled antibodies are shown. Medians were compared using Mann-Whitney test. (B) The distribution of K_D_ values is shown by subject. Each bar graph represents the median of the avidities of antibodies (represented by symbol) for each subject. The red dotted line represents the global median avidity of all SLE and control influenza-positive antibodies. (C) Avidities of 53 influenza-positive antibodies from SLE patients and 27 from controls were measured by SPR. Correlation between approximated K_D_ values from virus ELISA data and K_D_ values measured by SPR is shown. Black symbols are antibodies from SLE patients while blue are those from controls. Correlation was determined by Spearman’s correlation test. (D-E) Influenza-positive antibodies were serially diluted and tested in the standard HAI assay (D) and microneutralization (E) for functional capability. Medians were compared using Mann-Whitney test. (D) Minimum antibody concentration effective at inhibiting hemagglutination is plotted for each HAI-positive antibody. (E) Same as (D), except for microneutralization. Results are representative of at least three independent replicates.

To evaluate the binding capacities of anti-influenza antibodies from the SLE patients and controls, we tested all influenza-positive antibodies by ELISA to obtain half-maximal binding avidities (K_D_) to whole influenza virions (S1 Fig). Influenza-positive antibodies from SLE patients bound with significantly higher avidity (had lower K_D_ values) than control antibodies (Fig 1A). Furthermore, 6 out of 10 SLE patients (S1-6) had antibodies with median avidities above the global median compared to only 2 out of 8 controls (C1, 2) (Fig 1B). A more robust analysis of the affinities of 80 antibodies (53 from SLE patients and 27 from controls) using surface plasmon resonance (SPR), showed strong correlation with the virus ELISA K_D_ values across a range of affinities, verifying these results (Fig 1C, S3 Table). We conclude that the influenza-positive antibodies from some of the SLE patients have higher binding avidities to influenza virus than those from controls. This suggests that these high avidity antibodies from SLE patients would display improved protective capacities against influenza.

### Improved HAI and neutralization capacities observed for antibodies from SLE patients

Protective antibodies against influenza neutralize virions and prevent infection of host cells most often by binding and inhibiting the function of hemagglutinin (HA) and neuraminidase (NA) viral proteins [38]. The two most common *in vitro* methods for detecting such antibodies, the HAI assay and the microneutralization (MN) assay, were used to test the influenza-positive antibodies. Increased avidities correlate well with increased neutralization capacities (S1 Fig), thus antibodies from SLE patients had significantly better (lower) minimum effective concentrations for both HAI (Fig 1D) and neutralization (Fig 1E). Though not significant, there was also a tendency for a greater proportion of the SLE antibodies to be microneutralizing (p value = 0.07). We conclude from these experiments that functional capacities of individual influenza-positive antibodies are higher when from SLE patients.

The above results suggest that in the context of lupus, improved antibody responses can be generated against foreign antigens. However, with some SLE patients generating a greater proportion of particularly higher affinity antibodies (S1-6) and others generating a greater proportion of lower affinity antibodies (S7-10), it appeared that there were both high and low responders in the SLE cohort. SLE is a complex, heterogeneous disease and the patients may have different degrees of immune system abnormalities that may in turn influence their B cell antibody responses to different extents. It has also been reported that immunosuppressive treatment can affect the serological responses of patients to vaccination [17, 23] but we could not find a correlation between the median avidity of the anti-influenza antibody responses and immunosuppressive treatments of the SLE patients (S1 Table). We then examined other factors such as autoreactivity levels and gene usage that could potentially distinguish between the high and low avidity responders as well as provide insights into the foreign antigen-specific compartment of SLE patients.

### Similar overall levels of autoreactivity seen in influenza-positive antibodies from SLE and control subjects

One of the hallmark characteristics of SLE is the breakdown of primary B cell tolerance that leads to high numbers of polyreactive and self-reactive mature naïve B cells [2]. In addition, secondary tolerance is also disrupted in lupus, possibly leading to higher autoreactivity in the post-GC compartment. The observations that broadly neutralizing anti-HIV are polyreactive and self-reactive suggest that autoreactivity may be an advantageous characteristic for increased affinity [26, 29, 39]. However, SLE patients with high pre-existing titers of autoantibodies have lower serological responses to influenza vaccination [17], indicating instead that increased autoreactivity may be detrimental to a foreign-antigen response. Hence, we wanted to learn if the antibodies from SLE patients are more autoreactive than those from controls and whether the level of autoreactivity could differentiate between the high and low influenza vaccine responders. At the same time, analyses of the autoreactivity of the plasmablasts would allow us to evaluate the efficiency of secondary tolerance mechanisms operating in SLE patients during immune responses against foreign antigens.

**Fig 2:**
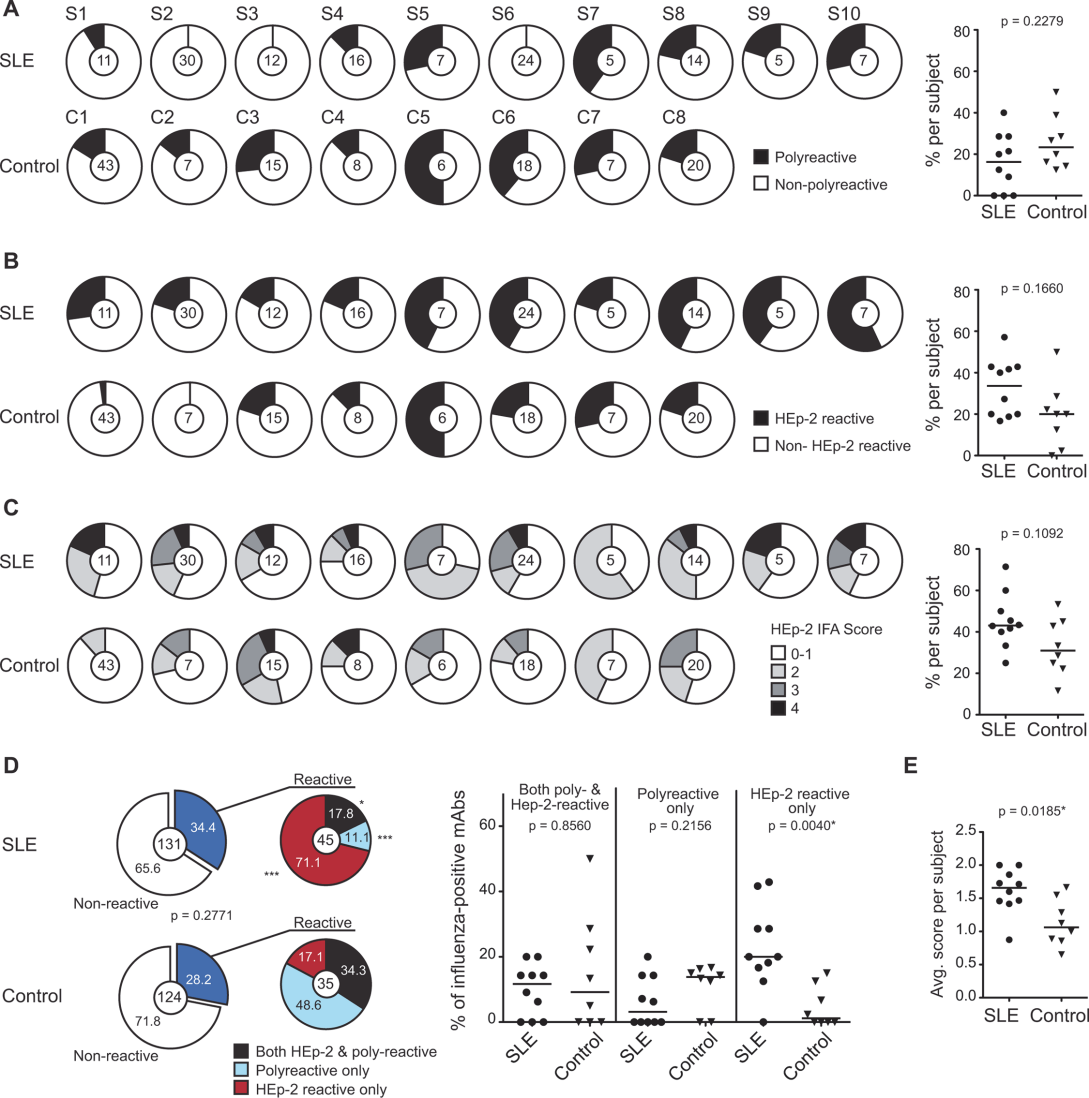
Poly- and self-reactivity of influenza-positive antibodies. Antibodies were tested for polyreactivity against dsDNA, LPS and insulin by ELISA and for HEp-2 reactivity by ELISA and IFA. (A) Pie charts represent the frequency of polyreactive and non-polyreactive influenza-positive antibodies for each individual. Numbers in the center indicate number of influenza-positive antibodies for each individual. Dot plot (right) summarizes the percent of polyreactive antibodies from the pie charts; each symbol represents an individual. (B) Similar to (A), except for HEp-2 ELISA reactivity. (C) Distribution of the HEp-2 IFA scores for each individual is shown and the dot plot summarizes the percent of antibodies with a score of 2 and above for each individual. (D) Distribution of antibodies with HEp-2- only, poly- only and both reactivities is shown for pooled influenza-positive antibodies in the pie charts. Values in pie chart segments are percentages. Statistical significance of the difference between the two cohorts was determined by the Chi-squared test (*p<0.05; **p<0.001; ***p<0.0001). Dot plots show the percent of each type of reactivity for each individual. (E) Average HEp-2 IFA score of all influenza-positive antibodies for each individual is plotted. Medians were compared by Mann-Whitney test in A-C, D (dot plot), E; and data are representative of at least three independent replicates.

To determine the level of polyreactivity in the plasmablast population of SLE patients, we tested all antibodies generated for reactivity against double-stranded DNA (dsDNA), insulin, and lipopolysaccharide (LPS) by ELISA. The frequency of polyreactive antibodies was similar for influenza-positive antibodies from SLE patients and controls (Fig 2A). There was also no difference in the frequency of polyreactivity for the influenza-negative antibodies (75 from SLE patients and 116 from controls) (S2A Fig). To determine if influenza-positive antibodies from SLE patients were self-reactive, we tested the antibodies for reactivity against human HEp-2 cells by ELISA and immunofluorescence assay (IFA). In each assay, although the frequency of influenza-positive antibodies with HEp-2 reactivity tended to be higher for SLE patients than for controls, it was not statistically different (Fig 2B and 2C). Consistent with this, when we combined the results of HEp-2 ELISA and IFA, we found that the percentage of total HEp-2 reactivity was higher for influenza-positive antibodies from SLE patients, although again not significantly (S2B Fig). Overall, the frequency of polyreactive and/or HEp-2-reactive influenza-positive antibodies was not significantly different between SLE patients and controls (Fig 2D). The frequency of self-reactive antibodies in the influenza-negative population was higher for SLE patients than controls (S2C and S2D Figs). This is consistent with past reports of increased CD27^hi^ B cells that reflect SLE disease activity [12, 13]. These data indicate that analogous to IgG memory B cells [5], the overall frequencies of polyreactive and HEp-2 reactive antibodies in the foreign antigen specific compartment of SLE patients are similar to that of controls.

Although not significant, the tendency for the percentage of HEp-2 reactivity by ELISA and IFA to be higher in SLE patients is nonetheless intriguing. To understand this better, we segregated the antibodies by their binding properties: HEp-2 ELISA reactive only, polyreactive only, and both HEp-2 ELISA reactive and polyreactive. While most self-reactive influenza-positive antibodies from controls were also polyreactive, the self-reactive influenza-positive antibodies from SLE patients tended to be only specific for HEp-2 antigens. This was observed both when antibodies were pooled and when segregated by individual (Fig 2D). This tendency was also observed for influenza-negative antibodies (S2E Fig). This indicates that the HEp-2 reactivity displayed by majority of the antibodies from controls may be a result of their inherent polyreactivity, thus leading to percentages of HEp-2 reactivity in controls that were skewed higher and not significantly different from that of SLE patients. In support of this, we found that the average HEp-2 IFA score of influenza-positive antibodies for each subject was significantly higher in the SLE patients than controls and there were more anti-nuclear antibodies in the SLE antibody set (Fig 2E, S2F and S2G Figs). These subtle differences between the self-reactive antibodies from SLE patients and those from controls suggest that while self-antigen specific B cells in SLE patients can survive secondary tolerance in the GC, selection against polyreactivity or other autoreactive epitopes in secondary lymphoid organs may be intact. To directly test if polyreactivity can be removed during foreign-antigen immune responses in SLE patients, we reverted four influenza-positive antibodies from SLE patients back to their predicted germline configuration and assayed their binding to polyreactive antigens. We found that two of the four antibodies became dsDNA reactive when expressed as unmutated, germline precursors, indicating that dsDNA reactivity of those two antibodies had been removed by somatic hypermutation (S2H Fig). Although strong conclusions cannot be drawn from analyses of only four antibodies, these data in combination with the increased HEp-2 reactivity suggest that autoreactive B cells in SLE patients may contribute to immune responses to foreign antigens.

### Higher percentage of autoreactive influenza-positive antibodies in most low responders

Although we could identify 9 HEp-2-reactive neutralizing antibodies (out of 51) from SLE patients, on the whole, autoreactivity did not correlate with increased influenza binding or neutralization capacity. Instead, SLE patients who had high avidity anti-influenza antibody responses (S2, S3 and S4) had low percentages of autoreactivity in their influenza-specific compartment whereas low responding patients (S8, S9 and S10) had high percentages of autoreactivity in their influenza-specific compartment (Figs 1 and 2). This observation is comparable to a past serological study that reported patients with high antinuclear antibody titers were low responders [17]. Our findings of the two SLE antibodies that displayed dsDNA reactivity when reverted to germline provide support that lower affinity binding to antigen is associated with autoreactivity. Within the controls, a similar relationship between high avidity response and autoreactivity levels was seen. Controls C1 and C2 who had high avidity anti-influenza antibody responses, had lower percentages of autoreactivity compared to the other controls. On the other hand, SLE patients S5 and S6 have high avidity anti-influenza antibody responses despite having high levels of autoreactivity. Hence, within our cohort with respect to influenza, it is inconclusive if autoreactivity is advantageous to the anti-influenza response. In the field of HIV, polyreactivity is proposed to improve the affinity of anti-HIV antibodies by giving them an advantage in binding low density epitopes on HIV through heteroligation. Unlike homotypic bivalent binding, heteroligation involves one antibody-binding site binding to the HIV envelope protein and the other cross-reacting to a low affinity site on another structure [26, 27]. On the contrary, the density of influenza HA and NA would allow for high avidity epitope-specific interactions by both antibody-binding sites without a selective advantage for autoreactive B cells. Autoreactivity could therefore be a property that is acquired through somatic mutations without a subsequent selective advantage against influenza.

### Biases in gene repertoire of influenza-specific plasmablasts from SLE patients

**Fig 3:**
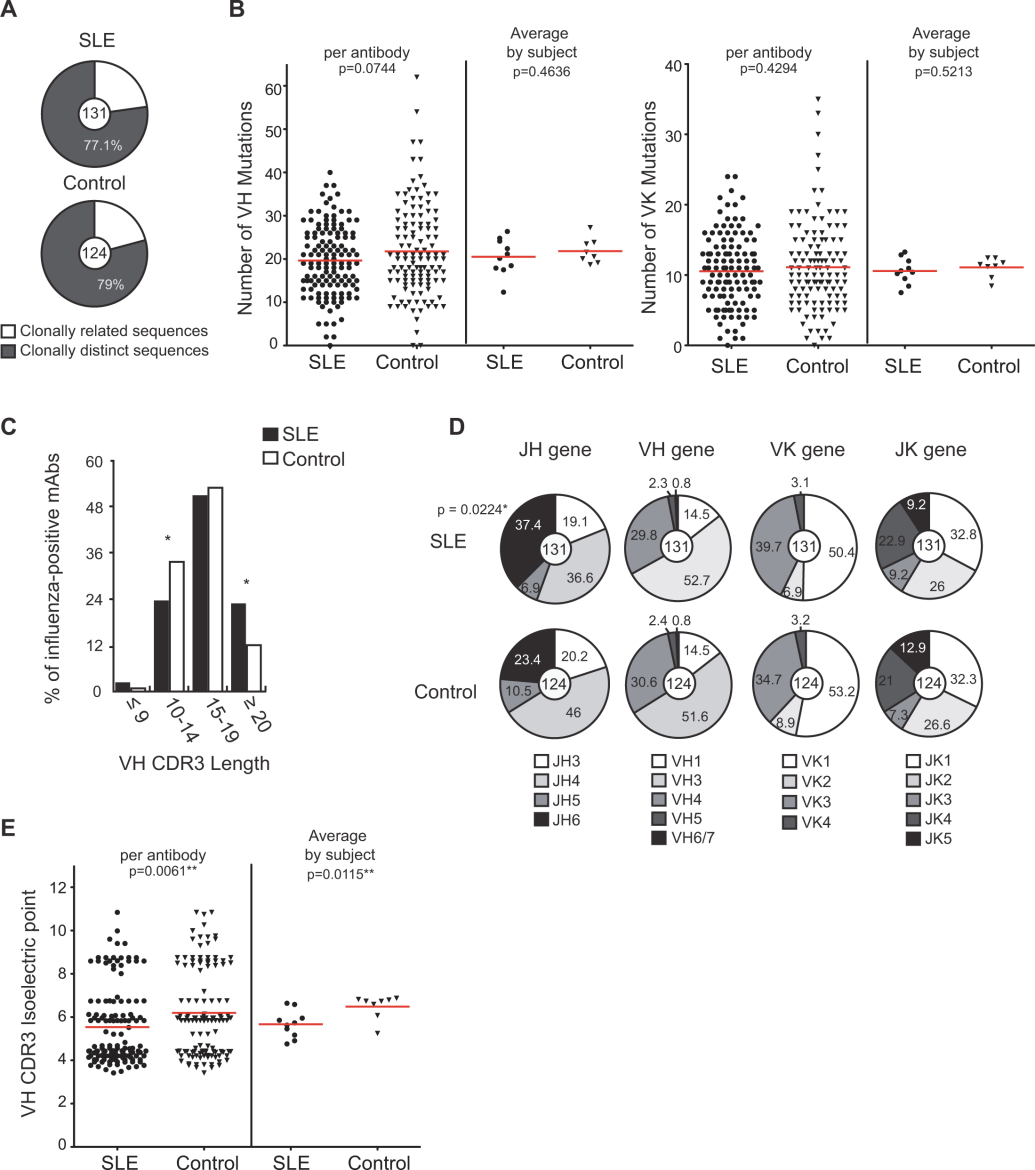
Heavy and light chain gene features of influenza-specific antibodies from SLE patients and controls. (A) Pie charts show the proportion of clonally related sequences in the influenza-positive antibodies from SLE patients and controls. (B) Number of somatic mutations in VH and VK chain gene sequences of influenza-positive antibodies is plotted. For each graph, in the left panel, each symbol represents an individual antibody, while in the right panel; each symbol is the average number of mutations of all the antibodies for each individual. (C) Proportion of influenza-positive antibodies with different variable heavy (VH) CDR3 lengths (number of amino acids). (D) Proportion of pooled antibodies with different joining region JH, VH, VK and JK gene identities. (E) Isoelectric points of VH chain CDR3 gene segments of influenza-positive antibodies from SLE patients and controls. The left panel shows each symbol representing an individual antibody, while in the right, each symbol is the average of all the antibodies for each individual. Means were compared in B and E using unpaired t-test. Chi-squared test was used to compare the frequency of the individual categories between SLE and controls (* p < 0.05) in C and D.

Next, we investigated the hypothesis that autoimmune B cell repertoires may have altered specificities that are otherwise removed from healthy individuals by intact primary tolerance mechanisms. Differences in the gene repertoire of influenza-specific plasmablasts could also reflect differences in B cell selection in the GCs between SLE patients and controls. We analyzed the variable heavy (VH) chain and kappa (VK) chain genes of influenza-specific antibodies from both cohorts to determine if there were differences in the plasmablast repertoires of SLE patients and controls. Although a greater number of subjects in the SLE cohort had clonally related sequences, overall, the proportion of clonally related sequences was similar between SLE patients and controls (Fig 3A). When we analyzed only unique clones, we observed similar results for the differences in influenza binding avidity between SLE patients and controls as in Fig 1. Since the clones did not skew the influenza binding data and each monoclonal antibody herein represents an individual plasmablast that was activated during the immune response against influenza, each antibody has equal weight in the analysis.

The number of somatic hypermutations in the VH and VK genes of influenza-positive antibodies from SLE patients and controls was similar when compared both by antibody and when averaged by subject (Fig 3B). This indicates that plasmablasts from both groups had similar levels of immune experience. Previous or repeated exposures to natural influenza infections or vaccinations greatly influence the immune response to a current influenza vaccination and the frequency of accumulated somatic mutations correlates with cross-reactivity to past influenza strains and thus with immune history[37]. We could not directly address the role of immune experience in the high and low responders within the SLE group and between the SLE patients and controls, as it is difficult to fully account for the lifetime history of influenza exposure of each subject. The similar levels of somatic mutations provide some evidence that immune experience to influenza is similar in the two groups. The average number of non-synonmous mutations in the CDR1 and 2, and framework regions was also not different for influenza-positive antibodies from SLE patients and controls.

In terms of the gene usage of influenza-positive antibody sequences, we found that SLE patients had a higher proportion of antibodies with long complementarity-determining region 3 (CDR3) segments (≥ 20 amino acids) in the VH chains (Fig 3C), as well as an increased usage of the joining (JH6) gene segment (Fig 3D). These two differences were not seen in the influenza-negative plasmablasts of SLE patients compared to that of controls. The predicted VH CDR3 isoelectric point (pI) was also significantly reduced for SLE influenza-specific antibodies (Fig 3E). During normal B cell development, long CDR3 segments and JH6 gene segments are counter-selected and their presence in the naïve B cell repertoire has been correlated with autoimmune repertoires [1, 40–43]. How these features of the antibody genes may influence the binding capacities of antibodies has yet to be investigated. Nevertheless, these differences in repertoires support a model in which the known break in primary B cell tolerance of SLE patients [1–3] leads to the production of a wide spectrum of autoreactive naïve B cells that are only partially controlled during foreign-antigen specific immune responses. Alternatively, it suggests that B cell selection in the GCs of SLE patients is altered, leading to unique repertoire properties in the post-GC compartment.

### Similar specificities seen in SLE and control antibodies

We then examined whether antibodies from SLE patients and controls differed in the targeting of influenza antigens. We found that similar proportions of antibodies from SLE patients and controls bound to recombinant HA (rHA) and to the conserved HA stalk versus the more strain-specific HA-head epitopes. Further, H1N1 and H3N2 rHA-specific antibodies from SLE patients and controls had similar levels of cross reactivity to multiple influenza strains when tested against a panel of rHA from past and diverse influenza strains using an antibody microarray (S3 Fig and S4 Table). These results suggest that the antibodies from SLE patients did not have differences in epitope targeting or breadth of variant influenza strains recognized and thus differential targeting of influenza antigens may not likely account for the improved influenza binding by SLE patient antibodies.

### Role of cytokines in high avidity antibody responses

**Fig 4:**
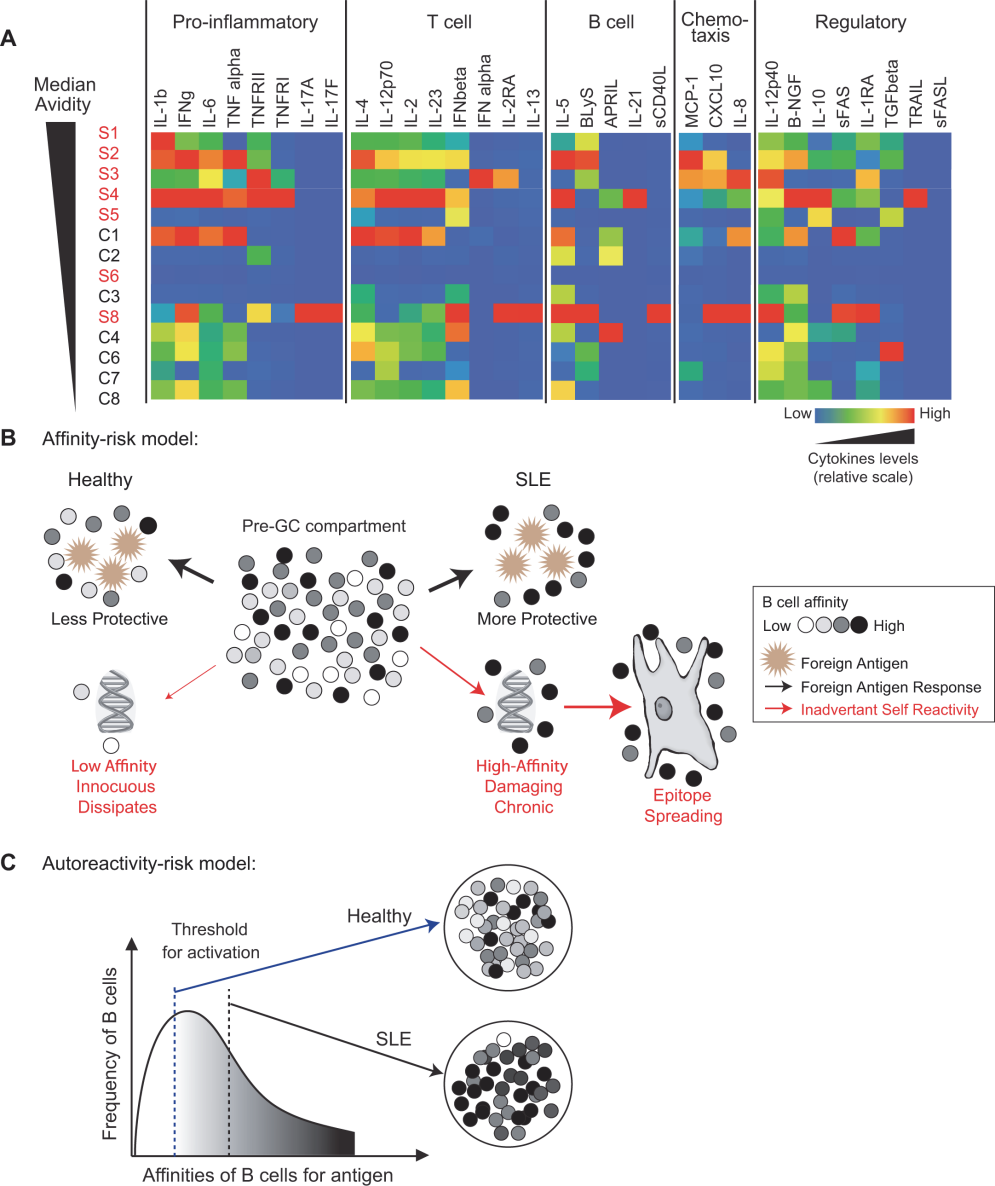
Distinct serum cytokine profiles seen in subjects with high avidity antibodies and models discussing the production of high affinity antibodies in context of autoimmunity. (A) The levels of various cytokines in the serum of subjects, measured by multiplex bead assay, are shown on a graded color scale. Cytokines are grouped according to their major roles in immune responses and subjects are ranked by the median avidity of each subject as shown in Fig 1D. Serum cytokine levels were normalized by the minimum (blue) and maximum (red) values within each dataset. (B) The affinity-risk model postulates that individuals who have the propensity to make high affinity responses will have higher risk for autoimmunity, especially if primary B cell tolerance mechanisms are also disrupted and the naïve B cell population is more self-reactive than normal. Thicker red arrows indicate greater probability of the event. (C) The autoreactivity-risk model proposes that anergy (or other factors) may be contribute to the increased activation threshold of self-reactive B cells in SLE patients so that only B cells with high enough affinity for the antigen are activated, thus skewing the response towards a higher affinity B cell response.

Another characteristic typical of SLE is the abnormal levels of various cytokines. Several cytokines are known to be dysregulated in SLE patients [44–47] and we reasoned that this could be a factor in determining the quality of foreign-antigen immune responses as germinal center reactions and B cell activation are greatly influenced by cytokines. Cytokines associated with SLE disease activity, such IL-2 and IFN- γ, profoundly affect T helper cell function and germinal center reactions, which then impacts antibody production by B cells [24, 48, 49]. To determine if serum cytokine levels could account for the high versus low avidity responses, we measured the pre-vaccination (day 0) levels of a panel of 32 cytokines by multiplex cytokine bead assay or ELISA. High levels of proinflammatory cytokines such as IL-1β, IFN-γ, IL-6, TNFα, and TNFRII; T cell cytokines such as IL-4, IL-12p70 and IL-2, as well as chemokines were seen in SLE patients (S1-4) who went on to produce a stronger anti-influenza response (Fig 4A). Interestingly, this same cytokine profile was observed for C1, the control individual with a large proportion of antibodies with high avidity (Fig 4A). The n value of subjects studied herein was not large enough to accurately access the correlation between the avidity of the anti-influenza response and the level of specific cytokines. In conclusion, these data suggest that pre-vaccination levels of key cytokines and chemokines that support GC reactions may influence the production of a high affinity response. Future experiments with a larger dataset would be needed to test this proposed hypothesis.

## Discussion

Influenza vaccination is routine for SLE patients and there have been many serological studies published that provide assessments of the antibody responses SLE patients make to the vaccine relative to healthy controls. Some studies showed that SLE patients have lower seroconversion rates while others reported no significant differences in serum antibody titers between vaccinated SLE patients and healthy individuals [14, 17, 20–22, 50]. The variability in the results of these studies possibly reflects diversity in the demographics of patient cohorts, disease heterogeneity and the varying degrees of patients’ lymphopenia. These issues, the lymphopenia of patients in particular, make serological data regarding the effects of lupus on the quality of B cell responses inconclusive, as it is difficult to distinguish between quantity of specific antibodies and quality of individual antibodies. Here, we specifically examined the quality of individual B cells activated in SLE patients by generating influenza-specific monoclonal antibodies from plasmablasts. We showed that higher binding avidities and neutralization capacities against influenza were observed for antibodies from SLE patients; with 6 of the 10 patients being the main contributors of high affinity antibodies.

The diversity seen in the responses of the SLE patients prevent us from making definite conclusions about the anti-influenza response generated in the context of lupus. Nonetheless, the generation of high affinity and more protective antibodies against influenza in SLE patients S1-S6 is interesting and it brings to mind the notion of autoimmunity as a selective advantage. Despite the ability of SLE patients to generate higher affinity antibodies, as suggested by our study, it has been reported that SLE patients have greater susceptibility to infectious diseases [51, 52]. This susceptibility is likely due, in parts, to reduced vitality resulting from autoimmune comorbidities, immunosuppressive therapy [17, 51], and disease-associated immune abnormalities such as leukopenia [12, 23, 53] that would impact the overall quantity of the immune response. Hence, the above contradiction suggests that an autoimmune tendency, without morbidity, could in fact mean improved protection against infections. The next implication of this interpretation leads us to a long-standing theory that autoimmunity developed as an unintended consequence of the host evolving to improve immune responses against pathogens. This hypothesis has been considered in the context of some infections. For example, a polymorphism of inhibitory receptor FcγRIIb protects individuals against malaria but increases susceptibility to systemic lupus erythematosus (SLE) [54–56] and the occurrence of HIV infections in SLE patients is disproportionately low [31–33].

There are several hypotheses that could explain the association between autoimmunity and improved immune responses and provide insights into the underlying abnormalities of autoimmune diseases. One hypothesis is that individuals who in general produce higher affinity antibodies have an increased risk for autoimmunity. In the event that tolerance for self is also disrupted, as suggested by past studies [2, 3, 57], then high affinity, pathological self-reactive antibodies could be produced at some frequency. These high affinity autoantibodies would be more damaging, causing a cascade effect where linked self-antigens are also targeted, leading to epitope spreading and eventual systemic autoimmunity (Fig 4B). In this case, natural selection for high affinity antibody responses, that would be beneficial against pathogens, could pose an autoimmunity risk.

Increased JH6 gene usage and long VH CDR3 segments, often seen in autoimmune repertoires, were enriched in the influenza-positive antibodies from SLE patients. Although we were not able to determine how these features allowed for enhanced influenza binding, it has been demonstrated by other studies that long CDR3 loops in anti-HIV and anti-influenza antibodies can extend into the receptor binding pockets of the antigen and contribute to the neutralization potency of the antibodies [58–61]. The observations on the repertoire differences support the hypothesis that due to impaired primary tolerance, the B cell repertoire available for immune responses against pathogens in SLE patients is less restricted. Consistent with this, another study showed that the IgG memory repertoire of SLE patients had altered VK2 gene family and VK3-20 usage [5]. It would be interesting to investigate if biases in the repertoire are seen in the antibody responses of SLE patients, relative to controls, for other pathogens.

Cytokines play important roles in immune system regulation and are an active area of investigation in lupus research. Several genetic polymorphisms associated with SLE affect cytokine signaling and alter cytokine profiles. For example, the risk allele of PTPN22 affects serum levels of interferon-α and TNF-α while the STAT4 risk allele affects serum levels of interferon-α [46]. We found distinct cytokine profiles (increased levels of IL-1β, IFN-γ, IL-6, TNFα, TNFRII, IL-4, IL-12p70 and IL-2) for subjects with higher avidity antibody responses (high responders) compared to those who had a lower avidity antibody response (low responders). These cytokines influence various immune cells, including B cells directly and indirectly through T cells and dendritic cells and therefore, would impact germinal center reactions during foreign-antigen responses. How the specific cytokines can contribute to the production of high affinity antibodies needs further investigation. Examining the cytokine profiles of a large cohort of healthy individuals in relation to their vaccination responses could provide insights for improving vaccine efficacy in low responders.

Yet another factor, not investigated herein, is the role of anergy. Human autoreactive naïve B cells have been reported to be anergic and intense stimulation is required for them to fully respond [40, 62]. It is possible that the high responders may have a high frequency of autoreactive lymphocytes that would be chronically stimulated, leading to anergy and reduced activation potential. Reduced activation potential would mean that the B cells require greater levels of stimulation to be activated. Hence, only the highest affinity B cells are activated and enter an immune response, resulting in the production of higher affinity antibodies (Fig 4C). This possibility is supported by our observation that reverting two SLE antibodies to germline configurations restored their DNA reactivity.

Genetic polymorphisms that are associated with SLE mediate risk of disease by altering immune regulation pathways, some of which overlap with pathways that are involved in immunity against pathogens. In fact, several studies have found that polymorphisms in viral RNA sensors such as Toll-like receptor 7 and MDA-5, also result in autoimmunity [63–66]. The link between viral immunity and autoimmune diseases provides insights into the evolutionary development of autoimmunity and also presents opportunities to study autoimmunity from a different perspective. The mechanistic basis of lupus in diverse human populations is at best poorly understood. Studying the generation of high avidity antibody responses in the context of dysregulated immunity could not only provide insights into mechanisms of immune responses against foreign antigens but also provide a new avenue for investigating the mechanisms of systemic autoimmune diseases.

In conclusion, we found that high affinity anti-influenza antibodies characterize the plasmablast response of SLE patients after influenza vaccination. It will be important to determine if similar observations are seen with other infections or vaccines, and in untreated SLE patients or unaffected family members.

## Supporting Information

S1 Fig Avidities and minimum effective concentrations of anti-influenza antibodies. Summary of the avidities and minimum effective concentrations in HAI and MN assay is shown for all influenza-positive antibodies (131 antibodies from SLE patients and 124 antibodies from controls). Each bar represents an antibody and grouping of antibodies is by subjects. Avidities were estimated by Scatchard plot analyses of ELISA data. Red lines indicate the global medians of all antibodies positive for the assay. (S-SLE; C-Control).
(EPS)

S2 Fig Poly- and self-reactivity of human monoclonal antibodies. (A-D, except B) Reactivity of influenza-negative antibodies. (A) Dot plot summarizes the percent of polyreactive antibodies in the influenza-negative antibody set from SLE patients and controls; each symbol represents an individual. (B) Dot plot summarizes the percent of HEp-2 reactive antibodies either by HEp-2 ELISA or IFA in the influenza-positive population of each individual. (C) Similar to (A), except for HEp-2 ELISA reactivity. (D) Dot plot summarizes the percent of influenza-negative antibodies with a HEp-2 IFA score of 2 and above for each individual. (E) Dot plots show the percent of each type of reactivity (poly- only, HEp-2 only or both) for the influenza-negative antibodies for each individual. Medians were compared by Mann-Whitney test in A-E and data are representative of at least three independent replicates. (FE) Distribution of the type of HEp-2 staining is shown for both influenza-positive and negative antibodies from SLE patients and controls. Representative images for each type of staining are shown within the legend. P values were determined by comparing SLE and control group using chi-squared test (*p<0.05; **p<0.001; ***p<0.0001). (G) Slide images of 18 SLE and 4 control antibodies with a score of 4 are shown. (H) Graphs show the reactivity of 4 influenza-positive antibodies from SLE patients (mutated and reverted versions) to dsDNA by ELISA. Each antibody was reverted to germline, synthesized and tested against dsDNA alongside its original, mutated version. Horizontal lines show the cut-off OD_405_ for positive reactivity. Data shown is representative of three independent replicates.
(EPS)

S3 Fig Cross-reactivity of rHA-positive antibodies. H1N1 and H3N2 recombinant HA (rHA)-positive antibodies were printed on a slide, each in triplicate, and incubated with various rHAs from previous and divergent influenza vaccine strains in an antibody microarray set-up. Signals were normalized to an internal control for each strain to obtain the relative level of binding of each antibody for the rHA. The relative level of binding of each antibody is shown on a color scale, with 100% (red) being the highest. An antibody was called cross-reactive if it had detectable binding to more one strain. The strains written in bold were in the vaccines received by subjects in this study. Results shown here are representative of three independent experiments.
(EPS)

S1 Table Information on the 10 SLE patients and 8 controls from whom plasmablasts were isolated 7 days after influenza vaccination and antibodies were generated. (PDF)

S2 Table Repertoire and reactivity of all antibodies expressed from SLE patients (206 antibodies) and controls (240 antibodies). (PDF)

S3 Table Binding kinetics data of 80 antibodies measured by surface plasmon resonance. (PDF)

S4 Table Array data on cross-reactivity of rHA-specific antibodies. (PDF)
