# Supplementary material for: High Affinity Antibodies against Influenza Characterize the Plasmablast Response in SLE Patients After Vaccination
Source: PLoS One. 2015 May 7;10(5):e0125618. doi: 10.1371/journal.pone.0125618 (PMC4423960; doi:10.1371/journal.pone.0125618)
Supplement: S1 Table — (PDF) [file pone.0125618.s004.pdf]

S1 Table. Information on the 10 SLE patients and 8 controls from whom plasmablasts were isolated 7 days after influenza vaccination and antibodies were generated

	Subject	Age	Sex[a]	Race[b]	Leuko-/ Lympho-penic[c]	ACR[d]	ANA[e]	Pattern[f]	dsDNA	Ro[g]	La[g]	nRNP[g]	UIL[h]	Rf[i]	Immunosuppresive therapy
**SLE patients**	S1	47	M	W	Y / Y	8	360	NS	-	+	-	+	-	-	Prednisone, Hydroxychloroquine, Mycophenolate mofetil
	S2	33	F	W	N / Y	6	1080	NS	-	+	-	+	-	-	Prednisone, Hydroxychloroquine, Mycophenolate mofetil, Methotrexate
	S3	48	M	W	Y / Y	5	1080	NH	-	+	+	-	-	-	Prednisone, Hydroxychloroquine
	S4	44	F	W	N / N	8	120	NH	+	-	-	-	-	+	Hydroxychloroquine
	S5	43	F	W	Y / N	7	120	NS	-	-	-	-	-	-	Prednisone
	S6	45	M	AA	N / N	5	120	NH	-	-	-	-	-	-	Prednisone, Methotrexate
	S7	31	F	W	N / N	6	180	NH	+	-	-	-	-	-	Prednisone, Hydroxychloroquine, Mycophenolate mofetil
	S8	50	F	W	Y / Y	6	3240	NH	+	+	-	-	-	-	Prednisone, Hydroxychloroquine, Azathioprine
	S9	34	F	W	N / N	4	360	NH	-	+	-	-	-	-	Hydroxychloroquine
	S10	51	F	AA	Y / Y	10	1080	NS	-	-	-	-	+	-	Prednisone, Hydroxychloroquine, Mycophenolate mofetil
**Controls**	C1	44	M	AA											
	C2	30	F	W											
	C3	29	F	W											
	C4	43	F	W											
	C5	39	F	W											
	C6	29	F	W											
	C7	51	M	W											
	C8	47	F	W											

[a]Male (M), Female (F). [b]Western European descent(W), African-American (AA) [c] Yes(Y), No (N) [d]American College of Rheumatology Criteria for Classification of Systermic Lupus Erythematosus [e]Anti-nuclear antigen (ANA) Titer (1/X) [f]NS: nuclear speckled; NH: nuclear homogenous [g]Autoantibodies against ribonuclearproteins by immunodiffusion [h]UIL (unidentified Line) in which autoantibody specificities are unknown [i]Rheumatoid Factor (anti-IgG)
