# Supplementary material for: High Affinity Antibodies against Influenza Characterize the Plasmablast Response in SLE Patients After Vaccination
Source: PLoS One. 2015 May 7;10(5):e0125618. doi: 10.1371/journal.pone.0125618 (PMC4423960; doi:10.1371/journal.pone.0125618)
Supplement: S2 Table — (PDF) [file pone.0125618.s005.pdf]

S2 Table. Repertoire and reactivity of all antibodies expressed from SLE patients (206 antibodies) and controls (240 antibodies)

Subject	Antibody	VH	JH	CDR3 (AA)	CDR3 Length (#)	#mutations	VK	JK	CDR3 (AA)	CDR3 Length (#)	#mutations	dsDNA	Insulin	LPS	Hep-2	Virus	Approx KD(M)[a]	rHA	HAI	MN
S1	2.AO6	3-23	4	AKFHFYGSGIGPEY	14	9	3-20	5	QQRDSSSIT	9	4	/	/	/	X	/		/	/	/
	2.BO6	3-73	4	FILPGYAMPR	10	16	3-20	5	QQYAGSPPIT	10	9	X	X	X	X	X	3.0E-07	/	/	/
	2.EO4	3-30-3	3	AALKLPIGYSFDI	13	28	1D-17	1	LQYNTYPWT	9	9	/	/	/	/	X	1.3E-10	/	/	/
	2.FO4	3-53	3	ATGGALGHRIYYDINGSQNFGFDV	24	40	1-16	4	QQYGSYPLT	9	16	/	/	/	X	/		/	/	/
	2.GO5	3-07	4	LRGGWHPDY	9	18	3-15	4	QQYNNWPRT	9	8	/	/	/	/	/		/	/	/
	3.AO5	3-73	4	IRQKGDTEMVIFDS	14	29	1-5	1	HQYFGPSTAT	10	17	/	/	/	X	X	2.6E-10	/	/	/
	3.EO1	3-11	5	ARRSNSAIPWFFDS	14	21	3-15	4	QQYNNWPLT	9	8	/	X	/	X	X	1.5E-07	/	/	/
	3.EO5*	4-31	4	ARGMLMDTDMIAFDQ	15	30	2-28	4	MQGIETPPT	9	13	/	/	/	/	X	3.5E-10	X	X	X
	3.FO6*	4-31	4	ARGMLLDTDMIAFDH	15	25	2-28	4	MQALQAPPT	9	11	/	/	/	/	X	2.5E-10	X	X	X
	3.2AO4	3-49	3	SKGGEGDAFDI	11	24	1-33	2	QQCVSLPYT	9	24	/	/	/	/	X	1.4E-09	X	X	X
	3.2AO5	5-a	5	ARHYWVGSNWFSAS	14	10	1-5	2	QQYNTYPCS	9	11	/	/	/	X	/		/	/	/
	3.2AO6	3-15	4	ATDRGDYYGSGTITGFLWVY	20	35	3-20	4	QQCGRSPPT	9	9	/	/	/	/	X	2.9E-09	X	X	X
	3.3BO6*	4-31	4	ARGMLMDTDMIAFDL	15	29	2-28	4	MQDIQAPPT	9	15	/	/	/	/	X	4.5E-08	/	/	/
	3.3DO6	4-34	4	ARQGNPHSSPLDY	13	34	1D-39	1	QQTDSTPHT	9	20	/	/	/	/	X	2.1E-10	/	/	/
	3.3GO3	1-69	6	ARSTYSGNFPRYYYYHGLDV	20	19	3-20	1	QQYGNSPPWT	10	11	/	/	/	/	X	1.6E-07	X	/	X
S2	1.EO2	5-51	3	ARQKYCSGGSCFGWHDAFDV	20	11	3-11	1	QQYGSSPPT	9	20	/	/	/	X	X	1.3E-06	/	/	/

	1.EO4	3-30	3	ARESGFCADDCARYAFDV	18	27	3-11	4	QHREKWPPGAT	11	22	/	/	/	/	/		/	/	/
	1.EO5	3-30	4	AKSGSFFGFDY	11	16	2D-29	4	MQSLQFPLT	9	8	/	/	/	X	/		/	/	/
	1.FO4	4-39	5	ARYALFDSGKNWFDP	15	32	3-15	2	QQYSNWPLCS	10	7	X	/	X	X	/		/	/	/
	1.GO1*	3-66	6	ARDRYSDGLGYYYYYYYGMDV	21	20	3-11	2	QHRFHWPQGS	10	14	/	/	/	/	X	7.2E-09	X	X	X
	1.2BO1*	3-66	6	ARDRYFDSAGRYYYYYYGMDV	21	18	3-11	2	QHRWRWPLCS	10	13	/	/	/	/	X	7.7E-07	X	/	/
	1.2BO2	1-69	3	ARYDYGGNSIFINPFDM	17	27	3-15	2	QQYNTWPSS	9	6	/	/	/	/	X	2.7E-09	X	/	X
	1.2CO1	4-39	3	ARVGGSAWKGGGADAFDF	18	12	3-11	4	QQRSNWPSLT	10	2	/	/	/	/	X	5.6E-09	X	/	X
	1.2CO3	4-39	6	ARAREGYNYLYGMDV	15	11	1-9	2	QHLDTWPRS	9	8	/	/	/	/	X	1.6E-09	X	/	/
	1.2EO3	1-69	4	ARWGGDASNRDFWSGPFDF	19	24	3-20	4	QQYGTSLP	8	14	/	/	/	/	/		/	/	/
	1.2EO5	4-59	4	ASGSDNGYMERRAYYLDN	18	22	3-20	3	QRYGTSPKFT	10	11	/	/	/	/	X	1.3E-08	X	/	/
	1.2FO3	4-39	6	ARQRAGWHYLYGMDV	15	19	1-27	1	QMYTGAPPWT	10	13	/	/	/	/	X	1.8E-09	X	/	/
	1.2GO1	3-53	6	ARDRYYDASGYYYYYYGMDV	20	21	3-11	2	QHRVKWPLCS	10	19	/	/	/	/	X	3.3E-07	X	/	/
	1.2GO5	4-34	3	ANQRRGYNSGYYYGPDAFDI	20	18	1-5	1	QQYDNYPWT	9	9	/	/	/	/	/		/	/	/
	2.BO4	1-02	4	ARSYDMRVVTVVSTGFDY	18	22	1-17	1	LQLNTYPWT	9	6	/	/	/	X	X	1.6E-09	X	/	X
	2.CO1*	3-66	6	ARDRYYDASGYYYYYYGMDV	20	28	3-11	2	QHCFNWPMCS	10	17	/	/	/	/	X	4.9E-08	X	X	X
	2.CO4	1-69	4	AGTRGYYGDYEPFDY	15	22	3-20	4	QQYGSSPRT	9	5	X	/	/	X	X	1.7E-09	X	/	X
	2.EO3	3-48	3	ARGKAGGSYLSDDALDI	17	21	1-5	1	QQYESYPWT	9	9	/	/	/	/	X	1.8E-09	X	X	X
	2.EO6	4-34	3	ATGRYSPNYFGSGSLAHAFDI	21	11	1-39	4	QQSYSTLALT	10	8	/	/	/	X	X	2.1E-07	X	/	/
	2.FO1	4-31	6	AREQYSGYEGGWGYYYYGMDV	21	2	1D-39	2	QQSYSTPRT	9	4	/	/	/	X	X	7.7E-07	/	/	/

	2.FO3	3-30	6	AKNSLPQYDDYRM EHPYFYGMDV	23	14	2-28	1	MQALQTPWT	9	4	/	/	/	/	X	8.1E-08	/	/	/
	2.FO4	3-21	4	ARDWGVKYADPEV FSD	16	26	1-5	2	QQYNSYSRT	9	12	/	/	/	X	X	1.7E-09	X	X	X
	2.FO5	4-04	3	ARGYYDILTGPSDA FDI	17	11	3-20	1	QQYGSSPRT	9	5	/	/	/	/	X	4.3E-09	/	/	/
	3.AO5*	3-09	6	AKDRIASSSLDYYY AYGMDV	20	16	1-33	4	QQYDNLPLT	9	11	/	/	/	/	X	3.4E-10	X	X	X
	3.DO2	1-69	6	ASTKRGESGRGLD V	14	26	1-33	2	QQYDNLLMSS	10	8	/	/	/	/	X	2.8E-10	X	X	X
	3.DO5	1-03	4	ARDPEIYYPDFRRV GILGYYFDC	23	26	3-20	3	QLYGSSPLFT	10	8	/	/	/	/	X	1.5E-09	/	/	/
	3.FO2*	3-09	6	AKDVAASSSMDYY YYSGMDV	20	16	1-33	5	QQYDNLPIT	9	8	/	/	/	/	X	3.5E-10	X	X	X
	3.GO1	1-18	3	ARAATAMGIDAFDI	14	21	1-39	1	QQTYSEPET	9	16	/	/	/	/	X	3.1E-07	/	/	/
	3.GO2*	3-09	6	AKDRSSSSSMDDY YYSGMDV	20	13	1-33	4	QQYDNLPLS	9	4	/	/	/	/	X	1.1E-09	X	X	X
	3.6DO1	3-11	4	VRCDSYSGYELDY	13	29	1-33	2	QQYDNLLMST	10	7	/	/	X	/	X	8.4E-08	X	/	/
	3.8CO3*	3-15	3	TNPTYGYYTEGGP ADGYPFDL	21	19	3-20	1	QQYGSSPWA	9	9	/	/	/	/	X	1.7E-10	X	X	X
	3.8FO5	3-30	4	AKEWDNSAYSLDY	13	15	1-6	4	LQDYNYPLT	9	11	/	/	/	/	X	2.9E-10	X	X	X
	3.8GO6	3-09	4	AKDPTGPIDSVGDF YFDS	18	16	3-20	1	QQYDSSPT	8	9	/	/	/	/	X	2.0E-10	X	X	X
	3.9CO5*	3-15	3	TNPTYGNYAEGGP AEGYPFDL	21	12	3-20	1	QQYGSTPWA	9	13	/	/	/	/	X	4.4E-10	X	X	X
	3.9GO6	3-20	3	ARGHEVSTSMDQH SDAFDV	19	10	1-5	1	QQYESLVG	8	5	/	/	/	/	X	4.1E-10	X	X	X
S3	2.AO4*	3-49	6	ARDQLELRGVIVRV GMDV	18	8	2-28	3	MQALQTPFT	9	7	/	/	/	/	X	8.7E-09	X	X	X
	2.CO2	3-15	4	TTVSGYCGGGICAF FDN	17	6	1-12	4	QQANSFPRT	9	4	/	/	/	/	X	8.6E-10	X	X	X
	2.CO3	3-07	6	AGDDTLWNGINA	12	23	3-20	2	QQFDRSPYT	9	20	/	/	/	/	/		/	/	/
	2.FO1*	3-49	6	ARDQLDLREVIVRV GMDV	18	9	2-28	3	MQALQTPFT	9	8	/	/	/	/	X	7.4E-10	X	X	X
	2.FO4	4-34	5	ARGMSYYGSGSYY FFYNWFDP	21	5	1-39	1	QQSNSSPRT	9	1	/	/	/	X	X	2.1E-08	X	/	X

	2.FO6	3-15	4	AKGYYYDTSGPYFFDS	16	14	3-20	2	QQYDRSMYT	9	3	/	/	/	/	X	5.5E-10	X	X	X
	2.GO1	3-66	3	ARERPTTTVTRTWGAFDI	18	19	3-11	5	QQRSDWPVT	9	7	X	X	X	X	/		/	/	/
	2.GO2*	3-49	6	ARDQLDLREVIVRVGMDV	18	13	2-28	3	MQALQTPFT	9	12	/	/	/	/	X	2.0E-09	X	X	X
	2.GO6*	3-48	4	ARDPFGYTDGHPFDS	15	25	3-15	1	QHYNDWPPWT	10	7	/	/	/	/	X	1.1E-08	X	X	X
	3.BO6*	3-48	4	ARDPFGYTDGHLFDY	15	19	3-15	1	QHLNNWPPWA	10	11	/	/	/	/	X	3.5E-09	X	X	/
	3.DO4	3-23	3	AKDDRGSGWTGAFDV	15	16	1D-13	2	HQFDDVPAT	9	10	/	/	/	/	X	8.5E-11	/	/	/
	3.DO6	3-09	6	AREPRGTADYYYYYGMDV	18	0	1-17	2	LQHNSYPYT	9	0	/	/	/	/	/		/	/	/
	3.FO5	3-49	4	SREMGDYYDSSGYYEDY	17	8	2-30	2	MQGAHWPYT	9	3	/	/	/	/	/		/	/	/
	3.2BO3	1-18	6	AREPNYGSGTYYYGMDV	17	9	2-28	3	MQGLQIPIT	9	7	/	/	/	/	/		/	/	/
	3.2BO4	3-30	6	SKDRYKIYFYGMDV	14	15	1D-39	3	HQGYNVPFT	9	17	/	/	/	/	X	7.5E-07	/	/	/
	3.2BO6	3-07	5	VTKLNDYGST	10	18	1-16	2	EQYNSYPNT	9	10	/	/	/	/	X	2.1E-10	/	/	/
	3.2CO1	4-39	6	ASGIGVLRYFDWFHAGMDV	19	0	2-24	5	MQATQFIT	8	0	X	/	/	X	X	4.0E-07	X	/	/
S4	2.AO2	3-07	4	AAGFGAHDY	9	16	4-1	1	QQYYNAPAT	9	13	/	/	/	/	/		/	/	/
	2.AO5*	3-09	4	AKDIAAGTHSSGYYNY	16	15	1-5	2	QEYNTFSSGT	10	17	/	/	/	/	X	1.1E-09	X	X	X
	2.BO3	1-02	3	ARVSVRYSDDAFDL	14	26	1-33	3	QQLFT	5	24	/	/	/	X	X	5.0E-09	X	X	X
	2.BO4*	3-23	4	AKLQGYTSSWFGTMDY	16	23	1-5	1	QQYDSLTWT	9	21	/	/	/	/	X	2.0E-09	X	X	X
	2.CO1	3-30	4	AKVALGYFEPDRFPISYYFDS	21	18	1-5	1	LQYSNHWT	8	7	/	/	/	/	X	4.6E-10	X	X	X
	2.CO3	3-23	5	AKSNEFVAVTGILDR	15	17	1-5	2	QQYSGYPYT	9	16	/	/	/	/	X	3.2E-08	X	/	/
	2.CO4	1-69	3	VLRGDEGAFHL	11	29	1-5	2	QQYYGFPYT	9	13	/	/	/	X	X	1.3E-09	X	X	/
	2.DO1*	3-09	4	VKDIAAGTHSSGYYNY	16	17	1-5	2	QQYNTFFSAT	10	12	/	/	/	/	X	1.2E-09	X	X	X

	2.DO6*	4-39	6	ARWAAGYYYFYGLDV	15	19	3-15	2	QQYDKWPGYT	10	5	/	/	/	/	X	4.5E-09	X	/	/
	2.EO2	3-30	6	TKVATWELDHFYGLDV	16	27	1-9	1	QQVNSYLWT	9	6	/	/	/	/	X	2.1E-10	X	X	X
	2.EO4	3-30-3	4	ARDESSGLIDY	11	40	2-30	1	MQGAHWPPIT	10	14	X	/	X	/	X	3.4E-10	X	X	X
	2.EO5*	3-23	4	AKLQGYTSSWYGTMDY	16	22	1-5	1	QQYDSLTWT	9	12	/	/	/	/	X	3.8E-09	X	/	X
	2.FO1*	4-39	6	ARRAGGYKYYYGLDV	15	31	3-15	4	QQYDDWLPLT	10	16	/	/	/	/	X	1.6E-09	X	/	/
	2.FO5	3-30	5	ARDRTGIGVAGTRWFDP	17	5	1D-39	2	QQSYSTPYT	9	8	X	X	X	X	/		/	/	/
	3.2BO2	1-18	4	ARTTIFGVVSDFDY	14	36	1-33	5	QQYDTFPIT	9	20	/	/	/	X	/		/	/	/
	3.2BO6	1-46	3	ARGDLDGSDV	10	15	3-20	5	QRYAMSTPVT	10	19	/	/	/	/	/		/	/	/
	3.2CO5	4-04	3	ARSEGLWTDGPRDAFDA	17	35	4-1	4	QQYYTTPHT	9	13	/	/	/	/	X	4.0E-07	/	/	/
	3.2CO6	1-69	6	ARVRDGHYYYTMDV	14	30	3-20	1	QQCSRSSTWT	10	11	/	/	/	/	/		/	/	/
	3.2DO1	3-23	3	AKGLSKAGSRSAFDI	15	7	4-1	2	QQYYSTPPMYT	11	1	X	X	/	X	/		/	/	/
	3.2DO2	1-69	6	ARDPMPREVPNTYYSALDV	19	37	3-15	1	QQYFSWPPA	9	13	/	/	/	/	X	5.8E-09	X	/	/
	3.2DO5	4-59	6	ARVKKYCSTSTCNYYADAMDV	21	21	3-15	3	QRYIGWPPTFT	11	13	X	X	X	X	X	3.5E-07	/	/	/
	3.2FO1	3-15	3	TTDTELRFLEWLPQAFDI	18	9	1-5	4	QQYHSFSLT	9	4	/	/	/	/	X	2.6E-07	/	/	/
	3.2GO1	4-59	6	ATSGLFSSPI	10	32	1-33	5	QQYDTFPIT	9	20	/	/	/	/	/		/	/	/
S5	2.AO2	4-b	5	AREDGSNSGSNYFDP	15	17	1D-39	2	QQSYSTPYT	9	5	/	/	/	/	/		/	/	/
	2.AO4	5-a	5	ARGGYSSDWYYWFDP	15	32	3-11	4	QQRRDWPT	8	22	/	/	/	/	/		/	/	/
	2.DO6	3-30	4	AKGRSNVPMDYIFDF	15	21	4-1	4	QQYYGAPIT	9	14	/	/	/	/	/		/	/	/
	2.EO3	1-02	4	ARDQGSY	7	1	1-33	4	QQYDNLPS	8	2	/	/	/	/	/		/	/	/
	2.FO3	3-23	4	AKSPFDSSGYMVN	13	22	3-15	1	QQYNYWPWT	9	12	/	/	/	X	/		/	/	/
	2.FO5	4-59	6	ARHSRPPSPFHYDSTMDV	18	31	3-15	2	QQSSYYYT	8	16	X	/	X	/	X	1.6E-10	/	/	/

	2.GO5	5-51	4	AQIFKWGSSWYQFDY	15	15	1D-39	4	QQSYSTPLT	9	13	/	/	/	X	X	3.1E-07	/	/	/
	3.2AO4	4-30-2	6	ARGVSQFSYGMDV	13	23	1-39	1	QQTYTTPA	8	18	/	/	/	/	X	4.6E-08	/	/	/
	3.2BO1	4-39	4	ARLYGSSSSGNY	12	27	1-5	1	QQYHSFPGT	9	8	/	/	/	/	X	3.1E-09	/	/	/
	3.2EO6	3-15	6	ATSIAFLSGYYNFYTGLDL	20	15	1-5	1	HQYKSSPRT	9	15	X	X	X	X	X	1.6E-07	X	/	/
	3.2GO5	3-74	4	VSQVFDFDNSGKSDN	16	26	3-11	3	QHRINWPFT	9	12	/	/	/	X	X	9.3E-11	/	/	/
	3.5CO1	3-30-3	4	ARAHQVAAAGTWTFDY	16	11	1-33	4	QHYENLPLT	9	2	/	/	/	/	X	1.5E-09	X	X	/
	3.5DO6	3-30	4	ARDAGYSVDWYPGAY	15	28	1-16	4	QQYDRYPLT	9	9	/	/	/	/	/		/	/	/
	3.5FO2	1-03	4	AVWGGQSRTYSLFDW	15	20	3-20	1	QYYSESSGA	9	18	/	/	/	/	/		/	/	/
S6	1.AO1	1-02	4	AREPVFLDY	9	21	3-11	1	QQRNNWPPTWT	11	7	/	/	/	X	X	1.3E-06	/	/	/
	1.AO3	4-30-4	6	AREKVYYASGISFTSGMDV	19	29	1-39	1	QQGFTTPWT	9	13	/	/	/	X	X	1.2E-06	/	/	/
	1.BO1	1-18	6	ARHYLQGVVVVDPYSHAMDV	20	20	3-20	2	QLYGSSRTT	9	14	/	/	/	X	X	2.3E-09	X	/	X
	1.DO2	3-15	4	SAGLGTSPLDS	11	28	2D-24	1	AHAAQYPWT	9	15	/	/	/	/	/		/	/	/
	1.EO1	1-2	4	TRTKYIAARPTFEY	14	21	1-39	1	QQNYNTPQT	9	12	/	/	X	X	/		/	/	/
	1.FO1	3-48	6	ARALFVVVPVTGGMDV	16	5	3-20	2	QQYGSSPPYT	10	4	/	/	/	X	X	3.7E-08	X	/	/
	1.FO3	3-15	5	TTDPGALYGRRWFDP	15	2	3-20	1	QQYGSSPRT	9	2	X	X	X	X	/		/	/	/
	1.2BO1	3-64	3	ARDPEDFFNSAGYSDI	16	33	1-16	2	QQYESYPCT	9	13	/	/	/	/	/		/	/	/
	1.2BO4	4-59	5	ARGKTIFGVVRNWFDS	16	11	1-13	5	QQFNSYPIT	9	9	/	/	/	X	X	1.4E-09	X	/	X
	2.AO1	5-51	3	ARTTCTSSTCYSLEANDAFDI	21	12	1-39	2	QQGHTTPQN	9	12	/	/	/	/	X	1.3E-08	X	X	X
	2.DO3	4-59	4	ARYCSNTRCSYMSFDY	16	19	1-39	2	QQSSDTLYS	9	15	/	/	/	/	/		/	/	/
	2.DO5*	4-59	6	ARERHYYETMDRIDYGMDV	19	13	3-20	5	QHYDISPSPRT	11	10	/	/	/	/	X	1.9E-09	X	X	X

	2.DO6	4-34	3	ASLRSGYQRRGFNV	14	7	3-20	2	HHYDSSPRVYT	11	4	/	/	/	/	/		/	/	/
	2.EO4*	4-59	6	ARERSYYETMDRIDYGMDV	19	14	3-20	5	QHYGRSPRT	9	12	/	/	/	/	X	1.6E-08	X	X	X
	2.GO4	3-53	6	ARSGVTIFGVVRPYGMDV	18	10	3-15	1	QQYNNWPPWT	10	5	/	/	X	X	X	1.8E-08	X	/	/
	2.2AO5	3-30	3	QLPTLASDV	9	44	2-30	2	MQGTHWPHT	9	15	/	/	/	/	/		/	/	/
	2.2DO1*	4-59	6	ARERSYYENMDKIDYGVDV	19	11	3-20	5	QQYLNSFRT	9	11	/	/	/	/	X	3.4E-08	X	/	/
	2.2DO3	4-30-4	4	ATSPSNYDNWNGFRLHFDS	19	19	3-20	4	QQYGTSPPWS	10	11	/	/	/	/	X	8.3E-09	X	/	/
	2.2EO1	3-21	5	ARALPREYRSNNWFDP	16	16	3D-20	2	QQYADFPYT	9	15	/	/	/	/	X	2.5E-10	/	/	/
	3.3AO6	4-61	4	ARTYYYDSSDYSQVLDYFDS	20	18	1D-39	2	QQSYNFRYT	9	11	/	/	/	X	X	3.1E-10	X	X	X
	3.3BO2	3-11	4	ARGTSSGRYFDY	12	18	3-15	4	QQYNDWPPFT	10	9	/	/	/	/	/		/	/	/
	3.3BO3	3-21	4	ARWAPDDYGDFPPHFDY	17	10	1-39	3	QQSYSTPPIT	10	3	/	/	/	/	/		/	/	/
	3.3BO6	4-b	4	ARVLYDFYSGTFFDA	15	29	3-20	3	QQYIASPPDIT	11	22	/	/	/	X	X	3.2E-07	X	/	/
	3.3CO1	3-48	6	ARDLDSDSSGDYYRGDHYYGMDV	23	18	3-20	2	QQYGSSPPYT	10	5	/	/	/	/	X	8.8E-08	/	/	/
	3.3CO3*	4-30-4	6	AGEQQHPQINYYALDV	16	19	1-39	1	QQIYSSFRT	9	12	/	/	/	/	X	1.0E-09	X	X	X
	3.3DO5*	4-30-4	6	AGEQHDLQINYYALDV	16	31	1-39	1	QQIYSNFRT	9	17	/	/	/	X	X	4.6E-09	X	X	/
	3.3EO2*	4-30-4	6	AGEQQYPQINYYSLDV	16	33	1-39	1	QQIYSGFRT	9	18	/	/	/	/	X	2.7E-09	X	X	X
	3.3EO6	1-02	4	ARDPSLDTSGYFDS	14	14	3-15	3	QHYNNWPPPIT	11	8	/	/	/	/	X	1.5E-09	X	X	/
	3.3FO1	1-69	6	SRVWEWRRLNRMDYCGMDV	19	30	1-39	1	QQSYITPVT	9	14	/	/	/	/	X	1.0E-09	X	X	X
	3.3FO3	3-53	6	ARVLTADYYYGMDV	14	5	1-39	4	QQSYSSPA	8	3	/	/	/	/	X	7.4E-09	/	/	/
	3.3FO5	3-49	4	ARDFAGYCTSTSCSHPYSHNYFDH	24	19	1-5	1	QQYDAYPRT	9	13	/	/	/	/	/		/	/	/
	3.3GO1	1-02	4	VREGGSSGYMTADY	14	25	1D-17	1	LQYDTYPWT	9	9	/	/	/	/	X	3.1E-10	/	/	/

	3.3GO4	3-23	3	AKDALFYYGSGEEAFDV	17	15	3-20	2	QHHGSSPYT	9	5	/	/	/	/	X	2.8E-10	X	X	X
	3.3GO5	3-73	6	SRPSYSSAWYLPNRMDV	17	25	1-33	2	QQYDNLPPFT	10	16	/	/	X	X	X	1.8E-07	X	/	X
S7	2.AO4	3-09	4	AAAFYGDPLKADY	13	16	1-5	1	QQYNNFPWT	9	16	X	/	X	X	X	3.1E-07	X	/	/
	2.BO3	3-30-3	6	AKDGGGYFYYGMDV	14	25	3-15	1	QQYNSWPRT	9	9	/	/	/	/	X	4.6E-09	X	/	/
	2.CO1	1-08	6	ARGPSSIAKHYMDV	14	16	1-33	2	QQYSNLPRRVI	11	22	X	/	X	/	/		/	/	/
	2.CO2	3-23	4	AKDRYGVGSNPLFDN	15	26	1D-39	5	QQSYSTPIT	9	13	/	/	/	/	/		/	/	/
	2.CO4	3-74	4	ARDALTSRSFDS	12	23	1D-39	2	QQSYSTPYT	9	16	/	/	/	/	/		/	/	/
	2.CO6	3-43	6	AKTYYDILTGPSYYGMDV	18	3	2-28	2	MQALQTPPYT	10	0	/	/	/	/	/		/	/	/
	2.DO2	3-23	4	AKDQVRVIPGTGYLDH	16	15	3-11	4	QQRSNWPPLT	10	8	X	/	X	/	X	2.1E-08	X	/	/
	2.DO5	3-15	3	TTSVKSGTVG	10	11	2-30	1	MQGTHWPWT	9	8	/	/	/	/	/		/	/	/
	2.EO2	3-30	6	AKDPGWRTQLPLNPDYYYGMDV	22	32	1-16	4	QQYDRYPLT	9	9	/	/	/	/	X	4.1E-07	X	/	/
	2.FO2	3-23	3	AKGAPPGSAFDV	12	25	2-30	2	MQGTHWPYT	9	6	X	/	/	/	/		/	/	/
	2.FO4	4-39	6	AHSGPYTIMDNFSYYALDV	19	24	3-15	5	QQYDNWPPIT	10	10	/	/	/	/	X	9.7E-10	X	X	X
	2.GO5	4-04	4	ARDYESAYSYGLLYY	15	20	4-1	1	QQYYSTPWT	9	15	/	/	/	/	/		/	/	/
S8	1.AO6	4-39	6	ARRAGGYLYFYGMDV	15	22	3-15	5	QQYNDWLPIT	10	12	X	/	X	/	X	4.3E-09	X	/	/
	1.DO1	1-18	4	VRNRYINSPFAN	12	24	1-9	5	QQVYIFPFT	9	9	X	X	X	/	/		/	/	/
	1.DO6	4-59	4	ARLSSRWGDGYTPAY	15	28	1-39	4	QQSYNTPLT	9	21	X	/	X	X	X	7.0E-07	/	/	/
	1.GO5	4-31	4	ARAPFNRRLVTLFGTYYFDT	20	26	3-20	5	QHYNSIPQNT	10	15	X	X	X	X	X	6.5E-08	X	/	/
	1.3EO2	3-49	6	TRDKSRDGATRVLYGMDV	18	10	4-1	4	QQFYTTPLT	9	7	/	/	/	X	/		/	/	/
	1.3FO5	4-59	5	ARRRVGQFVYHFDS	14	18	3-11	2	QRGSKWPPMYT	11	8	/	/	/	/	/		/	/	/

	1.3GO1	5-51	4	ARFDWPQFDY	10	3	3-15	4	QQYNNWPPLT	10	2	/	/	/	/	/		/	/	/
	2.AO1	3-11	6	ARDYYGSGNYLPYYYYAMDV	20	19	3-11	4	QERSNSFT	8	6	/	/	/	/	/		/	/	/
	2.BO3	3-53	6	AREATTVTTYGYYYYGMDV	19	2	3-15	2	QQYNNWPPPTLGNT	14	1	/	/	/	X	X	2.4E-07	X	/	/
	2.DO1	4-30-2	4	ARGYYSNYVPHFDY	14	12	1D-39	2	QQSSSTPYT	9	8	/	/	/	/	/		/	/	/
	2.EO1	4-39	6	ARHRIVARREFYYFGMDV	18	17	1-12	2	QQANSFPLT	9	14	/	X	/	/	/		/	/	/
	2.EO2	6-01	4	ARDRGYSGFGSFDY	14	15	1-12	2	QRTNSFPCT	9	12	/	/	/	/	X	4.5E-08	X	/	/
	2.FO2	1-69	5	ARDESRRVVAAAPFDS	16	24	3-20	2	QQYGSSPYT	9	7	/	/	/	/	/		/	/	/
	2.2AO3	1-46	4	ARDSNGVCLF	10	27	1-5	2	QQYKRLHS	8	15	/	/	/	/	/		/	/	/
	2.2CO1	3-07	3	ARGRAVDI	8	8	2-30	4	MQGTHWPPFT	10	13	/	X	/	X	/		/	/	/
	2.2DO3	3-07	4	ARVRGRGSYSGNYFDY	16	15	1-39	1	QQSYSTPWT	9	7	/	/	/	/	/		/	/	/
	2.2EO3	3-11	4	ARDRGEGTTTFDY	13	10	1-33	3	QQIFT	5	9	/	/	/	/	X	7.4E-09	X	X	X
	2.2EO5	1-03	6	AREKGVSGLFCRDGSCYGMDV	21	27	4-1	4	QQYYTPPLT	9	10	/	/	/	/	/		/	/	/
	2.2FO1	4-59	6	AKSLVRGVFYYYGMDV	16	10	1-39	1	QQSFSMPWT	9	10	X	X	/	X	/		/	/	/
	2.2FO3	1-03	6	ARAKEPMFRGLIDGYGVDV	19	18	4-1	4	HQYYSTPLT	9	10	/	/	/	/	X	7.6E-08	/	/	/
	2.2FO4	4-39	6	ARRRDGYKYYYGMDV	15	19	1-39	5	QQTYSPLPIT	10	5	/	/	/	/	X	9.4E-10	X	/	/
	3.DO2	3-21	3	ARVSPLSLIVGISGALDI	18	10	1-5	4	QHYNGYLT	8	5	/	/	/	X	X	2.5E-07	/	/	/
	3.EO1	4-39	4	ARPRYCSGGSCYSGLGQYHFDF	22	24	2-28	4	MQALQTPLT	9	6	/	/	/	/	/		/	/	/
	3.EO4*	1-46	3	ARDLLELLVYTYDSAGSSDAFDI	23	26	3D-15	4	QQYDTWPLT	9	9	/	/	/	/	X	4.4E-08	X	/	/
	3.FO5	3-48	4	ARGLFEGASRDCAGGRCSSAAFDI	24	14	1-33	2	QQYDNFPPT	9	7	/	/	/	/	X	6.5E-08	/	/	/
	3.GO4	4-59	6	GRGFYDSSSGPKNYGVDI	18	23	1-39	1	QQSYSTPRT	9	0	/	/	/	/	/		/	/	/

	3.2AO6	3-74	4	ARCLALSNIQCYHYFDQ	17	23	3-11	2	QQRSSWYT	8	12	/	/	/	X	X	1.8E-10	/	/	/
	3.2CO1*	1-46	3	ARDLLELLVYTYDSAGSLDAFDI	23	21	3-15	4	QQYDGWPLT	9	7	/	/	/	/	X	3.5E-08	X	/	/
	3.2CO2	3-43	6	AKDRSLRIFDRYGVDV	16	11	1-12	4	QQASSFPLT	9	18	/	X	/	X	X	1.9E-08	/	/	/
S9	3.AO3	4-30	4	ARGHHVSAYFDP	12	37	1D-39	2	QQSYSPPLYT	10	32	/	/	/	/	/		/	/	/
	3.CO3	3-64	4	VIRRGAATEY	10	37	3-15	1	QLYNNWPAWT	10	15	/	/	X	/	X	6.4E-07	X	/	/
	3.FO3	3-07	4	CVGGYAYW	8	12	1-17	1	LQYNIYPPT	9	5	/	/	/	X	X	6.9E-07	/	/	/
	3.FO6	3-07	6	AREVGFAGYWGSWTSYGMDV	20	24	1-9	3	QQLNTYPFT	9	6	/	/	/	X	/		/	/	/
	3.2BO3	3-53	5	VALQTYSYDTSGFTADGYYFDP	22	31	1-39	1	QQGYSMPWP	9	12	/	/	/	/	X	5.0E-10	X	X	X
	3.2EO4	1-02	6	ARSRAMDV	8	17	2D-29	4	MQTMQPLPA	9	12	/	/	/	/	/		/	/	/
	3.2GO6	3-53	4	ATNTVY	6	7	2D-29	1	MQRTQLPET	9	7	/	/	/	/	/		/	/	/
	3.3BO2	3-20	3	VRKAYGDAFDF	11	22	1D-39	5	QQSHSNPIT	9	26	X	X	/	/	/		/	/	/
	3.3CO1	3-23	4	ARRMIYGSGSFDS	13	23	1-9	1	QQLNSSPWT	9	11	X	X	X	X	X	4.3E-08	X	/	/
	3.3DO6	3-73	5	IRPDDHANPRT	11	21	1-5	2	QQYNTYLYT	9	10	/	/	/	/	X	5.8E-08	X	/	/
	3.3GO4	3-23	3	AKIRSTPGNHDASDI	15	11	1-5	1	QQYNSYWT	8	5	/	/	/	/	/		/	/	/
S10	1.BO2*	3-15	4	AHEASGALRD	10	34	3-20	3	QVFGGSPPFT	10	29	/	/	/	/	/		/	/	/
	1.CO6	3-48	3	AGDPDTPFIRVFEM	14	23	1-9	5	QQLNTYPPIT	10	13	/	/	/	X	X	3.2E-09	X	/	/
	1.DO3	3-30	6	ARSPASGYLDGDYFSWHYYSLDV	23	36	1-17	1	LQHNTYPYT	9	12	X	/	/	X	/		/	/	/
	1.EO1	4-39	5	AHSTPGYNSGWRPFDP	16	33	1-5	2	QQYHSYVYT	9	10	/	/	/	/	/		/	/	/
	1.EO2	4-59	3	ARDRTGMTDINAFDI	15	22	1-39	1	HQSYDTPQT	9	8	/	/	/	X	X	1.3E-08	X	/	/
	1.FO4	3-07	3	VRHNEARLATAYSGYDFDAYDV	22	23	1D-39	2	QQSYSALNS	9	23	/	/	/	/	/		/	/	/
	1.GO1	3-49	3	SRGGVSSGYDGLNALDI	17	11	1-39	1	QQSQSHYTPWT	11	11	/	/	/	/	/		/	/	/
	1.GO3	3-23	3	AKLRSSGWYAPIDI	14	28	4-1	2	QQYYSSPS	8	8	X	X	X	X	X	1.2E-07	X	/	/
	1.GO6	4-30-4	3	AREVIEPGDSDAFDI	15	23	3-15	4	QHYINQPLT	9	9	/	/	/	/	/		/	/	/

	2.CO3	3-30	5	ARDGNVLNGIDP	12	12	1-6	4	LQDYNYPPS	9	8	/	/	/	/	X	2.0E-07	X	/	/
	2.EO5	3-07	4	AREGGSYDWNDARY	14	13	1-16	4	QQYKTYPLT	9	8	/	/	/	X	/		/	/	/
	2.FO4	4-31	4	ARAAPYSDS	9	27	4-1	1	QQYHSTPPWT	10	16	/	/	/	/	X	1.4E-06	/	/	/
	2.FO5*	4-b	4	ARRGSGSYFSFDY	13	10	3-20	4	QHYGSSALT	9	1	/	/	/	X	/		/	/	/
	2.GO1*	4-b	4	ARRGSGSYFSFDY	13	14	3-20	4	QHYGSSALT	9	1	/	/	/	X	X	2.2E-07	X	/	/
	2.2AO1*	3-15	4	AHEASGALRD	10	29	3-20	3	QVFGGSPPFT	10	31	/	/	/	/	/		/	/	/
	2.2EO6	1-03	6	ARDRPDSSGYGSTGLDV	17	15	3-11	4	QQRTSWPLT	9	5	X	X	/	/	X	9.8E-07	/	/	/
C1	1.AO1	3-66	4	ATSPSAGY	8	13	1-5	2	QQYHSYPYT	9	22	/	/	X	/	/		/	/	/
	1.BO1*	3-48	4	AREEVVVAGTPFDS	14	18	3-15	1	HQYSGWPPWT	10	15	/	/	/	/	X	2.9E-09	X	X	/
	1.CO2	3-15	6	ATGGGSASGMYYYNGLDV	18	12	2D-29	2	MQSIQLPYT	9	10	/	/	/	/	X	3.4E-10	X	X	X
	1.CO5	4-b	3	VRDGSRYCVSGTCFSGDAFDV	21	32	3-NL5	1	QHYDNSPKT	9	70	/	/	/	/	/		/	/	/
	1.DO2	3-64	3	ARSRNQLLVSDALDI	15	7	3D-20	4	QQYGTSLLT	9	11	/	/	/	/	/		/	/	/
	1.DO5*	3-48	4	ANMRAWFWYDPADDIGH	17	18	3-15	1	QQYNKWPPWT	10	13	/	/	/	/	X	2.7E-09	X	X	X
	1.EO4	4-39	6	ARDRGGEEYYYGMDV	15	21	1D-39	2	QQGYSTPHT	9	19	/	/	/	/	X	2.8E-09	X	/	/
	1.FO2	4-39	6	AKINSGYEYYYGMDV	15	21	3-15	2	QQYNNWLYT	9	13	/	/	/	/	X	3.9E-09	X	/	/
	1.FO5*	4-59	5	ARSAQCTGDVCFGGPSWFDP	20	31	3-15	2	HQYNNWPYT	9	6	/	/	/	/	X	1.1E-08	X	/	X
	1.GO1*	3-48	4	ANMRAWFYYDPADDIGH	17	21	3-15	1	QQYNKWPPWT	10	9	/	/	/	/	X	4.9E-09	X	X	X
	1.2AO2*	3-48	4	VREAVIVDGMPFEY	14	35	3-15	1	HQYKGWPPWT	10	19	/	/	/	/	X	3.3E-09	X	X	X
	1.2AO6*	4-59	5	ARSAHCTDDVCFGGPSWFDP	20	15	3-15	2	HQYNNWPYT	9	4	/	/	/	/	X	5.1E-09	X	X	X
	1.2BO2	3-30	5	VKDPGDPWSGHFDP	14	29	3-15	2	HQYNNWPYT	9	11	/	/	/	/	/		/	/	/
	1.2BO5*	1-18	3	ARGFDYGDYRGRAFDV	16	15	1-33	3	QQSNT	5	6	/	/	/	/	X	1.4E-08	X	X	X

	1.2BO6*	4-39	6	ARVPDGYKYYYGMDV	15	28	3-15	5	QQYYNWTPIT	10	8	/	/	/	/	X	9.9E-10	X	/	/
	1.2DO3	3-15	4	TTASNPYNWNGPYFDY	16	10	1-5	2	QQYNVYSPMYT	11	3	X	/	X	/	X	4.0E-09	X	/	X
	1.2EO1	3-20	3	AREMRDPYDAFDL	13	28	1-39	5	QQSSTNPKT	9	22	/	/	/	/	X	1.6E-09	X	X	X
	1.2FO4	1-69	4	ARRYHSAPGYYFDS	14	32	3-15	1	QQYNDWTWT	9	12	X	/	X	/	X	2.2E-09	X	/	/
	1.2GO2	3-21	3	VKDRIQWTLRGAFDI	15	27	1-5	1	QHYEGYSWT	9	12	X	/	X	/	X	1.4E-10	/	/	/
	1.2GO4*	1-18	3	TRGFDYGDYRGRAFDV	16	15	1-33	3	QQSNT	5	9	/	/	/	/	X	5.4E-09	X	X	X
	1.3AO2*	3-23	3	AKNRGWQLGPDAFDI	15	23	1-13	5	QQFNSYPIT	9	6	/	/	/	/	X	5.0E-09	X	/	/
	1.3AO3*	4-39	6	ARRCGGYQYYYGMDV	15	17	3-15	5	QQYNNWAPIT	10	11	/	/	/	/	X	1.0E-09	X	/	/
	1.3BO1*	3-48	4	ANMRAWFWYDPADDIGH	17	21	3-15	1	QQYTKWPPWT	10	10	/	/	/	/	X	1.1E-07	X	X	/
	1.3BO2	4-59	4	ARFWREETYGSCFDY	15	20	1-39	1	QQSSTTPPWT	10	13	/	/	/	/	X	3.2E-09	X	/	/
	1.3CO1	3-30	5	VKDPGDPWSGWFDP	14	35	1-12	2	QQGFSFPYT	9	33	/	/	/	/	X	1.6E-09	X	X	X
	1.3CO4	4-30-2	6	ARVGDNGSSYGMDV	14	18	1-6	4	LQDYSYPLT	9	9	/	/	/	/	X	5.7E-08	X	X	X
	1.3EO3*	3-48	4	ANMRAWFWYDPADDIGH	17	25	3-15	1	QQYAGWPPWT	10	12	/	/	/	/	X	1.2E-08	X	X	/
	1.3EO5	4-39	6	ARQQEGYYYYYGMDV	15	20	3-20	3	QQYTSPGFT	9	5	/	/	/	/	X	1.2E-09	X	/	X
	2.BO2	1-02	3	ARAVYYDLTSDFCVDCYDAFDV	22	18	1-9	1	QQLNSYVVT	9	10	/	/	/	/	X	5.8E-10	X	X	X
	2.BO5	1-02	4	ARGPSISDGTENLFEF	16	30	4-1	4	QQYHKTPLT	9	30	/	/	/	/	/		/	/	/
	2.CO6*	3-23	3	VKNRGWQLGPDAFDS	15	47	1-13	5	QQFNSYPIT	9	35	X	/	X	/	X	2.4E-09	X	X	X
	2.DO5*	3-23	3	ARNRGWQLGPDAFAI	15	35	1-13	5	QQFNSYPIS	9	13	/	/	/	/	X	1.8E-09	X	X	X
	2.EO3*	3-15	4	TTVYYYESTAFITTGDY	17	18	4-1	4	QQHYTTPIT	9	15	/	/	/	/	/		/	/	/
	2.EO5*	3-48	4	VREEVIVGGIPFDY	14	31	3-15	1	HQYHDWPPWT	10	12	/	/	/	/	X	1.5E-09	X	X	X

	2.GO4*	3-23	3	VKNRGWQLGPDAFDI	15	34	1-13	5	QQFKSYPIT	9	16	/	/	X	/	X	1.4E-09	X	X	X
	2.2AO5	3-23	4	AKDQVGATSYYFDH	14	30	3-11	5	QQRNNWPTT	9	10	/	/	/	/	/		/	/	/
	2.2AO6*	3-15	4	TTVYYYESTAFITTGDY	17	18	4-1	4	QQHYTTPIT	9	15	/	/	/	/	/		/	/	/
	2.2BO2	1-69	5	ARSDWNDLFVL	11	29	4-1	4	QQYYTTPLT	9	6	/	/	/	/	/		/	/	/
	2.2BO3	1-24	6	ATHARVQSTYDQAYFYYYAMDD	22	7	3-20	3	QQYGTSPFT	9	8	/	/	/	/	/		/	/	/
	2.2CO3	4-59	4	AGAFLSNIGPYEY	13	32	1-5	2	QQCRSFSANT	10	17	/	/	/	/	/		/	/	/
	2.2CO4*	3-23	3	AKNRGWQLGPDGFDI	15	35	1-13	5	QQFNSYPIT	9	11	X	X	X	/	X	1.0E-09	X	X	X
	2.2CO6	1-69	6	ARQDIAVAGPNDYYYYNMDV	20	31	2-28	5	MQALQTPLIT	10	10	/	/	/	/	/		/	/	/
	2.2DO6	4-b	5	ARQSIYTGNHFPKFDP	16	17	1-13	4	QQFNSYPPEVT	11	7	/	/	/	/	X	2.0E-08	X	/	/
	2.2FO3	4-59	6	ARTMDTSGWQPYYYGMDV	18	17	3-20	2	QQYGSSPMYT	10	12	/	/	/	/	X	5.0E-08	X	X	X
	2.2FO4	3-30-3	4	VRGQDSSHYFDA	12	29	3-15	1	QQYDFWPT	8	16	/	/	/	/	X	2.5E-10	X	X	X
	2.2GO1*	3-23	3	AKNRGWQLGPDAFDI	15	33	1-13	5	QQFNSYPIT	9	18	X	X	X	/	X	1.4E-09	X	X	X
	2.2GO3	5-a	4	ARVYGDYALDH	11	30	1-9	1	QQLKSYPPT	9	22	/	/	/	/	/		/	/	/
	2.2GO5	3-74	5	VRGVMTYFDP	10	30	3-11	1	QHRGDGPPTWT	11	11	/	/	/	/	X	8.5E-09	/	/	X
	3.7BO1*	4-59	3	ARFHYYDNTGYYIDAFDI	18	16	1-5	2	QQYNSYPYT	9	6	/	/	/	/	X	2.9E-09	X	/	/
	3.7BO4	4-30-2	6	ARVGDNGSSYGMDV	14	18	1D-39	5	QQSYSNPIT	9	13	/	/	/	/	X	1.5E-08	/	/	/
	3.7BO5	3-23	3	AQEGYSSGRCGAYHV	15	23	1-5	1	QQYDNYSPA	9	19	X	/	X	/	X	7.5E-07	/	/	/
	3.7CO2*	4-59	3	ARFHYYDSSGFFIDAFDI	18	11	1-5	2	QQYNSYLYT	9	4	/	/	/	X	X	2.0E-07	X	/	/
	3.7DO2*	4-59	3	ARFHFYDSSGYYNDAFDI	18	12	1-5	2	QQYNSYPYT	9	10	/	/	/	/	X	4.2E-09	X	/	/
	3.7DO4*	4-59	3	ARFHYYDSSGYFIDAFDI	18	14	1-5	2	QQYNSYMYT	9	6	/	/	/	/	X	3.6E-09	X	/	/
	3.7DO5	1-46	3	ARAGYYYDSYGLDAFNI	17	8	3-11	1	QQRSNWPRT	9	0	X	/	X	/	/		/	/	/

	3.7EO4	3-11	6	ARGNYGFDV	9	9	3-20	1	QQYGSLSWT	9	9	/	/	/	/	/		/	/	/
	3.7FO2	1-46	3	ARGGVGPTWGYD<br>AFNM	16	37	3-11	2	QQYRFSSYT	9	15	/	/	/	/	X	1.2E-08	X	/	/
	3.7FO5	1-02	4	ARYTAGWYGDY	11	27	1-8	1	QQYYDFPRT	9	20	/	/	/	/	/		/	/	/
	3.7GO2*	4-59	3	ARFHFYDSSGFLN<br>DAFDV	18	27	1-5	2	HQYNSYPYT	9	22	/	/	/	/	X	1.8E-08	X	/	/
C2	2.BO4	3-23	4	AKQLLKGG	8	21	1-5	1	QQYVAYPWT	9	11	X	/	X	X	/		/	/	/
	2.EO2	1-18	4	ARDRSRIRFPFGY	13	5	3-20	1	QQYGTSPPT	9	2	/	/	/	/	/		/	/	/
	2.2BO2	3-23	3	TKRLPSWGAFEI	12	25	3-20	4	QQYPAAPLA	9	15	/	/	/	/	/		/	/	/
	2.2FO5	3-48	4	VREQYSSVWSVSH<br>FDF	16	23	1-39	2	QQSYSTPQCT	10	12	/	/	/	/	/		/	/	/
	2.2GO5	1-18	6	ARDLVQGAVSHYT<br>GLDV	17	25	3-20	1	QKYGSSTET	9	9	/	/	/	/	X	2.5E-08	X	/	/
	2.3AO6	4-61	6	AKMCSSSNCYSYF<br>YEMDV	18	24	2-30	5	MQASYWPRT	9	16	/	/	/	/	/		/	/	/
	2.3BO1	3-21	4	ARGDGSGNYLGEIL<br>FDY	17	21	1-5	2	QQYNSGYT	8	9	/	/	/	/	/		/	/	/
	3.AO3	3-48	4	ARVHVPYGDYIRTV<br>LDY	17	9	3-15	2	QQHNKWPPY<br>T	10	5	/	/	/	/	X	4.7E-09	X	/	/
	3.CO1*	4-04	4	ARLRNSPGTMFLD<br>Y	14	43	1-39	1	QQSYSTPWT	9	7	/	/	/	/	X	1.4E-07	/	/	/
	3.CO6	3-09	5	AKDAHDFRSGSGG<br>WFDA	17	23	3-15	5	QQCDNPIT	8	15	/	/	/	/	/		/	/	/
	3.DO6	4-59	4	ARDSTVFPRIFDS	13	13	3-20	1	QQCGSSPWT	9	10	/	/	/	/	X	2.7E-10	/	/	/
	3.EO6*	4-04	4	ARKRASTGTLYFDF	14	30	1D-39	4	QQTYSVPLT	9	24	X	/	X	/	/		/	/	/
	3.FO4	3-30	4	ARDYRHGLIDF	11	25	2-30	5	MQGTYWPPIT	10	14	/	/	/	/	X	4.5E-10	X	X	X
	3.GO3	3-74	4	AREPYHPPQFDY	12	16	1D-39	4	QQSYRSPPT	9	25	/	/	/	/	X	2.5E-09	X	X	X
	3.GO5	4-39	6	SRLQPYTNYGRSH<br>YYFYALDV	21	20	3-11	4	QERSNWPRLT	10	17	X	/	X	/	X	2.6E-07	X	X	X
C3	1.BO1	1-18	4	ARETLTYCSGGSCY<br>ARLSGNFDH	23	24	1-39	1	QQNYRTPWT	9	14	/	/	/	/	X	1.3E-07	X	/	/
	1.BO5	4-59	6	ARAGTWAELYGMD<br>V	14	6	2-28	1	MQALQTPLT	9	0	/	/	/	/	/		/	/	/
	1.CO4	3-30	4	TRGALRLAPGGTFT<br>Y	15	28	4-1	2	QQYYSLPYT	9	13	/	/	/	/	X	1.9E-07	/	/	/

	1.DO4	3-23	4	AARYCRYGDCYPYDY	15	29	3-20	2	LHYDSSPQS	9	10	/	/	/	X	/		/	/	/
	1.FO2	3-15	3	TWLLGYCSNGGCSDAFDL	18	14	4-1	2	LQYYNTPYT	9	8	/	/	/	/	/		/	/	/
	1.FO3	4-59	4	ARTWEYCTMTSCSPEGGTDF	20	25	1-33	3	QHYDSLGFA	9	8	/	/	/	/	/		/	/	/
	1.GO1	1-18	4	ARFKVPLYYDSSGTDY	16	26	1-6	1	LQDYTYPWT	9	7	/	/	/	/	/		/	/	/
	1.GO3	3-66	4	ARGLQDDARGVIRALYY	17	24	3D-15	1	QQYDNWPPWT	10	15	/	/	/	/	/		/	/	/
	1.GO5	3-30	6	AKEGSGSYYDTLGPNMDV	18	10	3-11	3	QQRSNWPPIT	10	3	/	/	/	/	/		/	/	/
	1.GO6	7-81	6	ARRDGNYVWYFDV	13	62	3-11	2	QLRSTWPPYT	10	15	/	/	/	X	X	8.4E-09	X	/	/
	1.2AO2	3-49	3	SREFTPVRAFDI	12	16	3-11	3	QHRTNWPLT	9	8	/	/	/	/	/		/	/	/
	1.2BO2	4-59	4	ARVDDFIWGTFDS	13	25	3D-15	1	QQYDGYPPWT	10	15	/	/	/	/	/		/	/	/
	1.2BO3	4-34	5	ARGFNPSGRGYNWFDP	16	14	1-39	1	QQSYIMPPT	9	12	/	/	/	/	X	3.0E-07	/	/	/
	1.2BO6	3-21	4	ASQGPDGDLNY	11	21	3-20	2	QQYGDSPLLT	10	8	/	/	/	/	/		/	/	/
	1.2DO5	3-07	4	VVTPRRDPFDY	11	7	2-30	1	MQAIHWPWT	9	4	/	/	/	/	/		/	/	/
	1.2EO4	3-33	4	ARPYCSSANCFPTYFDF	17	17	3-20	4	QQYSSSLALT	10	12	/	/	/	/	X	3.8E-08	/	/	/
	1.2GO2	3-21	3	ARDLTYGDYLKVFAFDI	17	14	1-33	5	QQYDNLPPSIT	11	9	X	/	/	/	/		/	/	/
	1.2GO3	3-53	4	ASGAPGRVAFDY	12	26	2-28	2	MQSLQTPYT	9	8	/	/	/	/	/		/	/	/
	2.CO1	3-15	6	TTDRITMVRGMLQYHYHSYDMDV	23	30	2-30	2	MQGTLWPT	8	12	/	/	/	/	/		/	/	/
	2.DO3	3-13	6	ARDRAYTGQNMDV	13	32	3-11	5	QQRTDWPSIS	10	15	/	/	/	/	/		/	/	/
	2.FO2	3-23	4	ATHKGHCTTGTCYFDN	16	13	3-15	3	QQYNNWPPVT	10	7	/	/	/	/	/		/	/	/
	2.3BO2	4-34	4	ARAGRVWGSYRFLDY	15	18	1-6	1	LQDYNYPWT	9	8	X	/	X	X	/		/	/	/
	2.3CO1	3-23	4	AKGVGRIFMVRGFYFDY	17	21	1D-39	3	QQSYSTPHT	9	9	X	X	X	X	X	2.6E-07	X	/	/

	2.3CO4	3-23	3	AKDLPQYGSTYWRGSFDM	18	10	1-9	5	QQLNNYPIT	9	1	/	/	/	/	X	1.8E-08	X	/	/
	2.3CO5	3-66	4	ATSPPRGGTYYFDY	14	9	3-11	4	QQRSQLVT	8	15	/	X	/	/	X	3.9E-07	/	/	/
	2.3DO6	4-59	6	ARDRGYSSGYKNYYYYGMDV	20	9	3-11	4	QLRSNSLT	8	7	X	/	X	X	/		/	/	/
	2.3EO6	1-03	4	ASPGKYQLAFDF	12	22	3-20	1	QQYGSSPPWT	10	21	/	/	/	/	/		/	/	/
	2.3FO2	3-30	6	AKLPQRLDEGDYYFDMEV	18	26	1-17	4	LQHSTYPLT	9	15	/	/	/	/	X	5.4E-10	X	X	X
	2.3FO3	3-66	4	AYDSSGYRTYQFDH	14	25	1D-39	5	QQSDTMPLT	9	14	X	X	X	X	/		/	/	/
	3.4AO5	3-30	4	AKDFWIAYYYGSGPLDN	17	18	3-20	2	QQYGSSFT	8	10	X	X	X	/	X	2.3E-07	/	/	/
	3.4BO3	3-30	6	AKVRIGLQSFGVYYGMDV	18	12	1-33	2	QQYDNLPVT	9	9	/	/	/	/	/		/	/	/
	3.4BO5	4-30-4	3	AREVDLARTTDAFDI	15	32	3-15	4	QHYNNWPLT	9	9	/	/	/	/	/		/	/	/
	3.4CO2	3-21	4	ARERGYSYGLDY	12	9	1D-39	2	QQSYITPHT	9	8	/	/	/	/	X	1.2E-08	X	/	/
	3.4CO4	5-51	6	ARQKVGATSYYYYNYGLDV	19	15	3-15	2	QQYDDWPLYT	10	5	/	/	/	/	X	8.0E-10	/	/	/
	3.4CO6	3-23	5	AKFPTIFGVVDS	12	14	2-28	1	MQARQTPRT	9	7	X	/	X	X	X	2.9E-08	X	/	/
	3.4EO4	3-74	4	ARETLRGYSGYVSFEY	16	21	1D-39	4	QQSYSIPLT	9	15	X	/	X	/	X	3.7E-07	X	/	/
	3.4FO6	4-39	5	ARRQYTNSPFDP	12	20	1D-39	5	QQGYSTPIT	9	17	/	/	/	/	X	4.5E-10	X	/	/
	3.4GO3	3-43	4	ATGGGSWYESGAGSLQYFDY	20	18	1D-39	3	QQSYNTPFT	9	11	/	/	/	/	/		/	/	/
C4	2.CO2	4-61	4	ARGRVPYSSSSGWF	14	21	1-39	2	QQSFTTPQT	9	9	/	/	/	/	X	9.7E-08	/	/	/
	2.CO3	3-30	4	ARDMGTATGTGFDH	14	20	3-11	2	HQRSNWPPMCT	11	6	X	X	X	X	/		/	/	/
	2.EO6	4-34	4	ARGPADVARYFDY	13	10	3-15	5	QQYNVWLRRIT	11	5	X	/	X	X	/		/	/	/
	2.GO3	3-23	6	VKGPTSYLLSGLDV	14	31	2-28	1	MQALELWT	8	6	/	/	X	X	X	4.5E-07	/	/	/
	2.2AO1	1-18	6	ATYSRAYFYGMDV	13	21	1-5	2	QQYNSYSCS	9	5	/	/	X	/	/		/	/	/

	2.2AO6	3-53	5	ARDRHCSGGSCYGA	14	8	2-28	5	MQARQTPIT	9	1	/	/	/	/	/		/	/	/
	2.2BO2	4-04	6	ARGPGEAVAGTLRWGPKEYHHNHRLDV	27	31	3-20	3	QHFGGSPLFT	10	11	X	X	X	X	/		/	/	/
	2.2BO6	3-15	6	MRDFYASGITRGDV	14	24	2-28	1	MQPLYTPRT	9	14	/	/	/	X	/		/	/	/
	2.2CO2	3-74	4	AAGTAY	6	28	2-30	2	LQGTHWPYT	9	16	/	/	/	/	/		/	/	/
	2.2CO3	5-51	4	ARQSIYDSSGYYYPFDY	17	23	3-20	1	QQYGDSPRT	9	12	/	/	/	/	/		/	/	/
	2.2CO4	3-33	4	VRSKELHENSGYFGY	15	31	1-5	1	QQYNSYWT	8	18	/	/	/	/	/		/	/	/
	2.2DO1	3-30	6	AKDRRRDSLPRYFDWLVVYNGMDV	24	27	2D-29	4	MQSLQVPLT	9	20	/	/	/	/	X	1.3E-08	X	/	/
	3.2CO4	3-53	5	ARGGGASYEFWSGYDL	16	21	4-1	1	QQYYNTPPT	9	7	/	/	/	/	/		/	/	/
	3.2FO3	3-15	4	STSGGSTWPPY	11	21	1-33	2	QQYDDLPYT	9	13	/	/	/	/	/		/	/	/
	3.2GO4	3-30	6	ASGGGGLYCSGRDCLYYALDV	21	25	1-33	2	QQYDDLPYT	9	13	/	/	X	X	/		/	/	/
	3.3AO4*	4-04	4	ARIAFVSSWYGYFDS	15	30	3-15	1	QQYDIWWT	8	5	/	/	/	/	X	2.1E-10	/	/	/
	3.3BO2*	4-04	4	ARVAYSGSWYGYFDF	15	21	3-15	1	QQYSVWWT	8	10	/	/	/	/	X	2.1E-10	/	/	/
	3.3BO3	4-61	4	ARNPPLDYSHSNRAYFFDY	19	18	1-17	4	LQHNTYPLT	9	8	/	/	/	/	X	2.7E-09	/	/	/
	3.3CO5	1-18	6	ARGEPRLVAPYALYSYHGMDV	21	16	3-11	4	QQRSDWPGELT	11	6	X	X	X	/	X	1.3E-07	/	/	/
	3.3FO4	3-15	4	STSGTTLGAAVEVF	14	54	4-1	4	QQFFSFPQT	9	30	/	/	/	/	X	3.4E-07	/	/	/
C5	1.AO3	1-02	4	TRGVGSPNYFDS	12	10	3-11	1	QQYGKSPRT	9	12	/	/	/	/	/		/	/	/
	1.AO4	3-23	5	AKDNGPSDGDWFES	14	38	2-24	1	MQVSHFPRA	9	13	/	/	/	/	/		/	/	/
	1.BO5	1-02	3	ARLLGIQLWLHAFDI	15	14	1-5	1	QQYNNFWT	8	5	/	/	/	/	/		/	/	/
	1.CO5	4-61	6	ARGARDFPRYVNYHMDV	17	25	2-28	2	MQGLQSPYS	9	11	/	/	/	/	X	5.6E-10	X	/	/
	1.DO2	3-23	4	AKTLKGGYDYGLDS	14	23	1-39	3	QQSYDTLT	8	14	/	/	/	/	/		/	/	/

	1.DO4	5-a	5	ARAAGGTLGWFDP	13	20	1D-39	4	QQSYSTPLT	9	11	/	/	/	/	X	1.1E-06	/	/	/
	1.EO2	3-07	3	ARGGRGAYYDFWSAYIDI	18	30	1D-39	2	QQSYHTPYT	9	16	/	/	/	/	X	2.5E-07	X	/	/
	1.FO1	4-30-4	6	AKTKTMSYFYTYMDV	15	33	3-15	5	QQHNDWPPIT	10	17	X	X	X	X	X	9.3E-09	X	X	/
	1.FO5	3-33	3	ARGHLGTTGAFDI	13	6	1-5	4	QQYKSYPLT	9	3	/	/	X	/	/		/	/	/
	1.FO6	4-61	5	ARSEGSSSFEWFDP	14	19	2-28	2	MQALQSPYS	9	9	/	/	/	/	/		/	/	/
	1.GO2	3-21	4	ARDRSSGWDPFDS	13	15	1-NL1	1	QQYYTTPPWT	10	5	X	X	X	X	X	5.7E-08	X	/	/
	1.GO5	1-03	6	SRDSLIRSLEWSNPVRNFHFSYMDV	25	20	1-5	5	QQYNSFHT	8	9	X	X	X	X	X	1.8E-07	X	/	/
	2.3GO1	3-21	3	ARDPYDSGWSVGRFGFDI	18	15	1-9	2	QQLNSYPRS	9	3	/	/	/	/	/		/	/	/
C6	1.AO6	3-13	3	AKALRGRRWLQSSDAFDI	18	36	1-39	1	QQSYTSRTRT	10	17	/	/	/	/	X	2.7E-08	/	/	/
	1.BO2	4-31	6	ARDRLTDFWSGYSVMDV	17	15	4-1	1	QQYYSDPT	8	12	/	/	/	/	/		/	/	/
	1.CO1	3-30	4	ARVASMILVDIKYYFDY	17	17	3-15	1	QQYNEWPRT	9	4	X	X	X	X	X	2.4E-09	X	/	X
	1.CO3	3-23	6	AKDITEYTYGLGFGYFYDYGLDV	23	15	1-9	3	QQLNSSPFT	9	8	/	X	X	X	/		/	/	/
	1.CO4	3-30	6	AKVLYDILTGYRYGMDV	17	10	1-12	3	QQANSFPFT	9	5	X	X	X	X	X	1.4E-07	X	/	/
	1.DO2	1-69	4	ANMSTVTTSDL	11	38	2-28	2	MQALQIPFA	9	12	/	/	/	/	/		/	/	/
	1.DO5	3-30	3	ARERNFFHFGAFDL	14	21	1-5	2	QQYDTFSS	8	10	/	/	/	/	/		/	/	/
	1.EO1	3-30	4	AKDQRGSSGWERGHGFDY	18	7	2-28	1	MQPLQTPWT	9	0	/	/	/	/	/		/	/	/
	1.EO2	1-69	4	ARDEYYDSRGYPVGGHFDN	19	15	3-11	1	QQRSNWPRT	9	5	/	/	/	/	X	9.6E-07	/	/	/
	1.EO4	1-69	5	ASPPPESSGYLGWFDP	16	27	3-15	2	QQYYNWPPRYT	11	9	X	/	X	/	X	1.1E-06	/	/	/
	1.FO5	1-46	3	ARAGYYYDSYGLDAFNI	17	8	1-9	2	QQLNSYPYT	9	1	/	/	/	/	X	2.6E-07	/	/	/
	1.GO3	3-09	4	VKGRGGVYTGYGPRYLDY	18	15	1D-39	3	QQSYSSPRT	9	11	X	X	X	/	X	1.9E-07	X	/	/

	1.GO4	3-09	4	AKAAKPGPARAPFDS	15	20	2-28	4	MQALETPLT	9	9	/	/	/	/	X	4.6E-07	X	/	/
	1.GO5	4-31	4	ARVRIEYYYWSSRPNKPGAFDY	22	10	1-33	4	QQYDNLPLT	9	2	/	/	/	/	/		/	/	/
	1.GO6	4-61	4	ARTNYYDSSGY	11	9	1D-39	4	QQSYSIPLT	9	6	/	/	/	/	X	5.0E-07	X	/	/
	2.AO5	7-04-1	4	ARVKYGSGSYIY	12	21	4-1	2	LQYFTNTQS	9	16	/	/	/	/	/		/	/	/
	2.BO1	3-09	3	TKPYLMRSTFDI	12	17	3-20	2	QQYETAPYT	9	12	/	/	/	/	X	3.2E-07	X	/	/
	2.CO1	3-15	4	VTDFSGRRPY	10	18	1-5	3	QLYNDYSGFT	10	8	/	/	/	/	X	2.0E-08	X	X	X
	2.CO2	3-74	4	ASAKGGYSYGNFDY	14	9	3D-15	2	QHYNNWPPRYT	11	2	/	X	X	/	X	1.1E-06	/	/	/
	2.CO3	3-64	3	GKDLVFGAAVPDVGPDGCDI	20	71	1D-39	4	QQTYSIPLT	9	11	/	/	/	X	/		/	/	/
	2.DO1	1-18	5	ARDRGSILGSDPWFDP	16	17	2-30	1	MQGTHWPLT	9	7	/	/	/	/	X	3.4E-08	X	/	/
	2.DO2	3-30-3	6	ATRSVGATGYLSWGPKDYSYNDVDV	25	38	1-17	1	LQHNSYPWT	9	10	X	X	X	X	X	9.5E-08	X	/	/
	2.EO1	1-69	4	ARSPDSYDGSGYPFPH	16	26	4-1	3	QQYYSSPPT	9	11	/	/	/	/	/		/	/	/
	2.EO4	3-13	3	ARAPGDDIGAFDI	13	18	1-5	1	QQYNSYFTWT	10	13	/	/	/	/	/		/	/	/
	2.FO2	1-18	4	AGIRTYSGYDYFDY	14	6	1-12	1	QQANSFPVT	9	7	/	/	/	/	/		/	/	/
	2.FO4	3-23	4	AKGGDYYALGTYLEQFDY	18	9	4-1	4	HQYYSTPLT	9	4	/	/	/	/	/		/	/	/
	2.FO6	3-23	4	VQLGNYDY	8	35	2-30	3	MQGSYWPPFT	10	18	/	/	/	/	/		/	/	/
	2.GO4	4-39	4	ASSRSNSYGHDYFDY	15	10	1-6	1	LQDYNYPWT	9	7	/	/	/	/	/		/	/	/
	2.2BO5	3-07	6	AREGQTVTPPKYHYGMDV	18	11	3-20	2	QLYGSSANT	9	10	/	/	/	/	/		/	/	/
	2.2CO6	3-23	4	AKKSPNDYDHY	11	21	1-5	1	QQYNSYSGT	9	15	/	/	/	/	X	1.0E-08	X	X	X
	2.2DO3	3-09	3	VKATTYQYDRDGFDV	15	6	4-1	4	QQYYTTPLT	9	5	/	/	/	/	/		/	/	/
	2.2EO5	1-69	6	AKVRTPGPPTLPEMEGLFGDDYLADLCYGMDV	32	27	2-28	1	MQSLQTPQT	9	13	/	/	X	/	X	5.6E-10	X	X	X

	3.AO2	3-07	6	ARDPYNRNEAGYGMDV	16	27	2-30	2	MQGTKWPYT	9	8	/	/	/	/	/		/	/	/
	3.AO6	3-48	3	AREVDTYGDEGDAFDV	16	6	2-30	2	MQGTHWPGYT	10	2	/	/	/	/	/		/	/	/
	3.BO2	4-34	4	ARGVTMLRGARPDVRQPKYDY	21	14	1-39	1	QQSYITPRT	9	8	X	X	X	X	X	2.5E-07	X	/	/
	3.BO4	1-03	5	ARAGYSSVEEFGWFDP	16	20	4-1	2	QQYYSTPQT	9	5	/	/	/	/	/		/	/	/
	3.BO5	1-18	5	ARDPGYCTTTDCYWIWFDS	19	10	4-1	2	QQHYSTPYT	9	3	/	/	/	/	/		/	/	/
	3.BO6	1-08	5	ARGLRGQFLVLGGNWFDP	18	24	2-24	2	MQTTQFPYT	9	34	/	/	/	/	/		/	/	/
	3.CO4	3-33	6	GKDSLEVAVMTGTFGLDV	18	15	3-11	1	QQRVNWPLT	9	8	/	/	/	/	/		/	/	/
	3.CO5	3-64	4	ARDRYCSSTNCYAPLSY	17	2	4-1	4	QQYYSTPLT	9	1	/	/	/	/	/		/	/	/
	3.DO1	3-30	4	VRPGRHCSSTSCYAHFDS	18	14	4-1	3	QQYYNTPLT	9	8	/	/	/	/	/		/	/	/
	3.DO2	3-21	4	AREWGHYDTRGEFDF	15	23	3-15	4	QQYNNWPPLT	10	5	/	/	/	/	/		/	/	/
	3.FO3	3-66	4	VRQSYDGGVWGHYDS	15	43	4-1	4	QQFYNNPST	9	10	/	/	/	/	X	9.2E-08	/	/	/
C7	2.BO4	3-66	6	AGDPILRNGMDV	12	24	3-11	4	QQRHSWPRT	9	16	/	/	/	/	/		/	/	/
	2.DO2	3-09	3	AKGENNWNDPDAFDI	15	0	1-16	4	QQSNEKPLT	9	10	/	/	/	/	X	1.4E-06	X	/	/
	2.FO3	3-15	4	ATRGPRGMYFNY	12	16	3-20	1	QQYVTSPWT	9	4	/	/	/	/	/		/	/	/
	2.GO5	3-07	3	ARQWELLEWVDAFDI	15	5	1D-39	2	QQSYSTPYT	9	1	/	/	/	/	/		/	/	/
	2.GO6	1-24	4	ATDYNY	6	19	1-16	4	QQYSSYPLT	9	13	/	/	/	/	/		/	/	/
	3.AO1	3-48	4	AREAYYGSGSYKF	13	26	1-33	3	QQYDSLPFT	9	12	/	/	/	/	/		/	/	/
	3.AO5	3-33	6	ARDGFSDYDTYYNGIDV	17	17	3-11	2	QQRFNGLCS	9	6	X	X	X	X	X	6.5E-09	X	X	X
	3.CO2	4-61	4	ARDNVVSLGRFGEFALYIDY	20	22	1-39	5	QQSYGIPLT	9	19	/	/	/	/	X	5.0E-08	X	/	/
	3.DO3	1-46	4	ATVPTGAGDY	10	47	1-5	3	QLYNDYSGFT	10	10	/	/	/	/	X	7.1E-07	X	X	/
	3.DO6	1-69	4	ARAAYILTTGTLHF	14	21	3-15	4	QQYNEWPSLT	10	9	/	/	/	/	/		/	/	/

	3.FO2	4-59	4	ARGGRPMGSSSFYYFDY	17	29	1D-39	4	QQSHTSPLT	9	27	/	/	/	/	X	3.9E-07	/	/	/
	3.GO2	3-74	4	IRVECC	6	21	1-5	4	QHYYDSPLT	9	17	/	/	/	/	/		/	/	/
	3.2AO2	4-34	4	ARGYDYVWGSYRRRPYYFDY	20	0	1-5	1	QQYNSYSLT	9	0	X	X	X	X	X	2.3E-07	X	/	/
	3.2AO3	3-33	4	ARGREYCGSSTCYDVFDY	18	20	1-9	1	QQLNSYPRT	9	14	/	/	/	/	/		/	/	/
	3.2BO4	3-48	4	ARSHRVTSNVPDY	13	19	3-11	2	QQRSNWPFMYA	11	7	/	/	/	/	X	1.7E-10	/	/	/
C8	1.AO6	1-08	5	ARGKSTITTYWFDP	14	8	4-1	2	QQYYSNPYT	9	9	/	/	/	/	X	6.0E-07	/	/	/
	1.BO2	3-30	4	AKDLVRYFEWVGAD	14	13	2-29	1	MQGIHLPWT	9	11	X	/	/	X	X	5.9E-08	X	/	/
	1.BO4	4-59	6	ARLGFGEVFSFKRYYYYGLDV	21	17	3-20	3	QQYGSSVLT	9	19	X	X	X	X	X	3.2E-07	X	/	/
	1.DO5	4-59	5	ARGGSSGRHGWFDP	14	29	3-20	4	QQYGSLPLT	9	16	X	/	/	/	/		/	/	/
	1.FO3	3-72	6	ARGSNYYYGMDV	12	8	2-30	1	MQGIDWPWT	9	15	/	/	/	/	/		/	/	/
	1.FO6	3-72	4	VRWTQGACDC	10	17	3-11	1	QHRSNWPET	9	3	X	/	/	/	/		/	/	/
	1.GO1	4-39	6	ARIAVEGLDV	10	28	1-5	4	QEYNSYSLT	9	2	/	/	/	/	X	1.0E-06	/	/	/
	1.GO2	1-2	5	GRDGRNIVEIRAAVSDR	17	49	3-15	3	HQYYKWPS	8	13	X	/	X	/	/		/	/	/
	1.2AO2	4-61	4	ARGPSLVDF	9	11	1D-39	2	QQSYNSPYT	9	8	/	X	/	X	X	9.5E-08	X	/	/
	1.2AO4	3-23	6	VKDRGTMFRGAVYGMDV	17	6	1-17	2	LQHNSYPYT	9	1	X	X	/	/	X	4.0E-07	X	/	/
	1.2BO5	4-61	4	ARDLVRLTSTGPTYYFDS	18	27	1-33	3	QHYDSLPLT	9	17	/	/	/	/	/		/	/	/
	1.2BO6	3-49	5	TRDRYKWNYLVNE	13	10	1-39	1	QQSYSIPWT	9	9	/	/	/	/	X	3.0E-07	/	/	/
	1.2CO3	3-15	4	TVDDSAYISQIDY	13	16	2-28	1	VQTLQTWT	8	6	/	/	/	/	X	3.5E-07	X	/	/
	1.2DO3	3-07	4	ARLGIISGSGLNDY	14	18	1-16	1	QQYISYPPT	9	12	/	/	/	/	/		/	/	/
	1.2DO5	3-23	4	AKDKIVGSIWVYYFDY	16	3	3-20	1	QQYARSPWT	9	8	/	/	/	/	X	2.5E-07	/	/	/
	1.2DO6	3-23	4	AKREGGWYQHYFDY	14	15	3-20	2	QQYGSSPYT	9	3	/	/	/	/	/		/	/	/
	1.2EO6	4-59	4	ARVVTVAGLVN	11	9	1-9	4	QQLNSYPRT	9	2	/	/	/	X	X	2.9E-07	X	/	/
	1.2GO2	5-51	4	VRGASSNLFDY	11	15	1-5	1	QQYNTYSWT	9	12	/	/	/	/	X	3.4E-07	X	/	/

	1.2GO3	1-02	4	VREGPVRGLTPPDY	14	17	3-11	4	QQRSDWPLT	9	7	/	/	/	/	X	6.1E-07	X	/	/
	2.BO6	3-15	4	TTGSGTIQLKLGGEY	15	20	4-1	3	QQYYSIPFT	9	18	/	/	/	/	/		/	/	/
	2.CO3	3-23	6	VKRGWFGELFGNYGMDV	17	39	1-9	4	QQLESYPLT	9	10	X	/	X	/	X	8.2E-08	X	/	/
	2.CO4	3-23	4	ANTYASRNNIKFLGY	15	34	1-39	1	LQTYSDPWT	9	19	/	/	/	/	X	1.1E-06	/	/	/
	2.EO1	4-39	4	ARGPTSRWAHFDY	13	17	3-11	2	QQRSFWPPRYT	11	2	/	/	/	/	/		/	/	/
	2.EO6	3-23	6	TKVDSSPFFDYGMDV	15	24	1-17	1	LQHNSYPWT	9	13	/	/	/	/	/		/	/	/
	2.FO2	1-69	4	SRDSNGGGAYDS	12	23	3D-15	4	QQYNSWLRA	9	10	/	/	/	/	X	4.9E-09	X	/	X
	2.FO6	3-09	5	TKDVGITTAAIGS	13	20	3-15	1	QQYNHWPLT	9	15	/	/	/	/	/		/	/	/
	2.GO1	3-49	4	VSDDESDTPVDFDY	14	38	2D-29	4	MQNMQLPPT	9	14	/	/	/	/	/		/	/	/
	2.GO2	3-23	6	AKDRGRAAVVDYYYSMDV	18	17	1-8	1	QQYYSYPRT	9	8	X	X	X	/	/		/	/	/
	2.GO5	4-59	6	ARGNWGPPNYPYYYGMDV	18	30	2-28	4	MQAVQGLPVT	10	19	/	/	/	/	/		/	/	/
	3.AO2	3-30	4	AKDLLPGWAAGGRYYFDN	18	25	1-16	4	QQYDRYPLT	9	9	X	X	X	/	X	3.0E-07	X	/	/
	3.CO4	4-59	5	ARDRSPLGNWLDP	13	21	1-NL1	4	QQYYTTPLT	9	11	/	/	/	/	/		/	/	/
	3.FO3	3-21	6	ARAPYDSSGYDYIFYYDAMDV	21	10	1-5	1	QQYKSYSRT	9	3	/	/	/	/	X	1.4E-06	/	/	/
	3.GO4	4-04	4	AREPKAQASRFDF	13	36	1-33	4	QRSDDLPLA	9	13	/	/	X	/	X	9.0E-07	/	/	/
	3.2AO6	3-74	3	AKSYKMNYSAFDI	13	32	1-12	4	QQSNDYPLT	9	18	/	/	/	/	X	1.1E-09	X	/	X
	3.2BO6	3-23	3	ARDRPKMRTTRRVSDVLSGPKGRGGSYVFDI	31	39	4-1	4	QQYYSPPLT	9	13	X	X	X	X	/		/	/	/
	3.2EO5	3-11	4	ARGSMRFSEWSPLGF	15	22	3-20	2	QQCGSPPFT	9	17	/	/	/	/	X	1.4E-07	X	/	/
	3.2FO6	4-61	4	ARERGYSGGGSLGH	14	32	1-5	2	QYYDSFSMYT	10	11	/	/	/	/	/		/	/	/

*X indicates reactivity in ELISA; *next to antibody names refers to clonally related members*

[a]K_D values estimated by Scatchard plot analyses of virus ELISA data
