# Supplementary material for: High Affinity Antibodies against Influenza Characterize the Plasmablast Response in SLE Patients After Vaccination
Source: PLoS One. 2015 May 7;10(5):e0125618. doi: 10.1371/journal.pone.0125618 (PMC4423960; doi:10.1371/journal.pone.0125618)
Supplement: S3 Table — (PDF) [file pone.0125618.s006.pdf]

S3 Table. Binding kinetics data of 80 antibodies measured by surface plasmon resonance

Antibody	KD (M)	ka (1/Ms)	kd (1/s)	Chi2
S1 3.EO5	1.44E-10	6.28E+06	9.01E-04	3.21
S1 3.FO6	2.11E-10	3.81E+06	8.02E-04	3.8
S1 3.2AO4	9.18E-11	1.91E+05	1.75E-05	2.99
S1 3.2AO6	1.67E-08	6.86E+04	1.15E-03	2.91
S2 2.BO4	2.48E-10	4.58E+05	1.14E-04	3.83
S2 2.EO3	5.20E-10	8.01E+04	4.16E-05	3.42
S2 2.FO4	1.08E-10	1.29E+05	1.39E-05	2.87
S2 3.AO5	3.22E-11	1.60E+06	5.16E-05	3.03
S2 3.FO2	4.84E-11	3.53E+06	1.71E-04	2.92
S2 3.GO2	1.95E-10	1.09E+06	2.11E-04	3.11
S2 3.8CO3	2.87E-11	8.08E+05	2.32E-05	3.2
S2 3.8FO5	5.01E-11	1.71E+06	8.59E-05	3.09
S2 3.8GO6	6.43E-11	3.51E+06	2.26E-04	3.09
S2 3.9CO5	8.78E-11	4.75E+05	4.18E-05	3.2
S2 3.9GO6	4.84E-11	2.06E+06	9.98E-05	3.05
S3 2.AO4	4.03E-09	8.14E+04	3.28E-04	2.79
S3 2.CO2	8.64E-10	8.33E+04	7.20E-05	3.24
S3 2.FO4	3.54E-09	1.89E+04	6.70E-05	3.71
S3 2.FO6	2.37E-10	2.01E+05	4.76E-05	3.07
S3 2.GO2	2.71E-09	1.38E+05	3.75E-04	2.98
S3 2.GO6	1.38E-09	1.19E+05	1.63E-04	2.98
S3 3.BO6	1.58E-09	1.44E+05	2.28E-04	3.44
S4 2.AO5	1.40E-10	2.66E+05	3.73E-05	2.69
S4 2.BO3	9.60E-11	1.55E+04	1.49E-06	2.47
S4 2.BO4	1.46E-10	4.11E+05	5.99E-05	3.56
S4 2.CO1	9.67E-11	1.69E+05	1.63E-05	2.8
S4 2.CO3	5.09E-09	1.62E+05	8.25E-04	3.91
S4 2.CO4	1.03E-09	1.01E+05	1.04E-04	2.76
S4 2.DO1	3.94E-10	2.12E+05	8.34E-05	2.65
S4 2.EO2	4.91E-10	5.54E+05	2.72E-04	4.22
S4 2.EO4	2.51E-09	6.51E+05	1.63E-03	2.98

S4 2.EO5	4.44E-10	4.18E+05	1.86E-04	3.53
S4 3.2DO2	8.03E-10	5.02E+05	4.03E-04	3.27
S5 3.5CO1	1.08E-09	1.81E+05	1.94E-04	3.57
S6 1.BO1	1.41E-10	2.85E+05	4.03E-05	2.99
S6 2.AO1	2.58E-08	5.29E+04	1.37E-03	2.71
S6 2.DO5	5.95E-10	1.25E+05	7.42E-05	2.96
S6 2.EO4	1.34E-08	1.93E+05	2.58E-03	2.76
S6 2.GO4	6.48E-09	8.42E+04	5.46E-04	3.19
S6 3.3AO6	1.06E-11	2.67E+06	2.84E-05	3.89
S6 3.3CO3	1.72E-10	1.06E+06	1.83E-04	3.4
S6 3.3EO2	1.94E-10	1.13E+06	2.20E-04	3.21
S6 3.3EO6	5.03E-10	2.85E+05	1.43E-04	2.92
S6 3.3FO1	2.72E-10	6.63E+05	1.80E-04	3.4
S6 3.3GO4	1.25E-11	2.23E+06	2.78E-05	3.21
S7 2.BO3	2.13E-10	8.58E+04	1.83E-05	2.85
S7 2.DO2	2.60E-09	1.25E+04	3.26E-05	3.05
S8 2.2EO3	1.66E-09	3.23E+04	5.37E-05	2.84
S8 3.EO4	2.17E-09	2.93E+05	6.35E-04	2.88
S8 3.2CO1	3.70E-10	2.65E+05	9.81E-05	2.81
S9 3.2BO3	4.68E-10	7.19E+05	3.37E-04	4.73
S9 3.3CO1	3.63E-10	7.65E+04	2.78E-05	2.69
S9 3.3DO6	2.99E-09	3.12E+05	9.33E-04	3.01
C1 1.CO2	1.16E-09	5.59E+05	6.48E-04	2.98
C1 1.DO5	1.51E-09	2.69E+05	4.07E-04	3.58
C1 1.2AO2	1.14E-09	3.16E+05	3.60E-04	3.58
C1 2.DO5	4.34E-10	2.09E+05	9.08E-05	3.49
C1 2.EO5	1.24E-09	5.57E+05	6.91E-04	3.96
C1 2.GO4	1.41E-10	1.05E+05	1.48E-05	2.79
C1 2.2CO4	1.37E-10	2.19E+05	3.01E-05	3.41
C1 2.2FO4	1.84E-10	7.46E+05	1.38E-04	3.23
C1 2.2GO1	2.54E-10	1.45E+05	3.68E-05	2.82
C1 3.7BO1	1.50E-10	2.40E+05	3.59E-05	3.34
C1 3.7DO2	3.32E-10	2.32E+05	7.70E-05	3.42
C1 3.7DO4	2.10E-10	1.92E+05	4.03E-05	3.82
C1 3.7FO2	2.52E-10	7.02E+04	1.77E-05	3.39
C1 3.7GO2	7.66E-10	1.45E+05	1.11E-04	3.38

C2 3.AO3	1.49E-11	9.01E+05	1.34E-05	3.25
C2 3.FO4	3.30E-10	1.77E+05	5.86E-05	3.7
C2 3.GO3	1.90E-10	9.26E+04	1.76E-05	3.37
C2 3.GO5	1.35E-09	3.20E+05	4.32E-04	3.77
C3 2.3FO2	1.75E-09	7.11E+05	1.24E-03	3.74
C3 3.4CO2	1.14E-09	1.53E+05	1.75E-04	2.86
C4 2.2DO1	1.35E-09	4.38E+04	5.92E-05	3.19
C6 2.CO1	5.92E-09	4.17E+04	2.47E-04	3.93
C6 2.DO1	2.43E-09	3.29E+04	8.00E-05	2.64
C6 2.2EO5	1.08E-10	2.37E+05	2.56E-05	3
C7 3.AO5	9.84E-09	1.52E+04	1.50E-04	2.84
C8 2.FO2	5.78E-10	6.07E+04	3.51E-05	3.63
C8 3.2AO6	2.03E-11	1.44E+06	2.92E-05	4.13
