# Supplementary material for: High Affinity Antibodies against Influenza Characterize the Plasmablast Response in SLE Patients After Vaccination
Source: PLoS One. 2015 May 7;10(5):e0125618. doi: 10.1371/journal.pone.0125618 (PMC4423960; doi:10.1371/journal.pone.0125618)
Supplement: S4 Table — (PDF) [file pone.0125618.s007.pdf]

S4 Table. Array data on cross-reactivity of rHA-specific antibodies

H1N1 rHA-specific antibodies

Antibody	A/New Caledonia/20/99	A/Solomon Islands/03/06	A/Brisbane/59/07	A/South Carolina/1/18	A/Puerto Rico/8/34	A/USSR/90/77	A/Texas/36/91	A/California/04/09
S1 3.2AO4	17.3808	6.44266	1.89806	5.81614	6.4423	1.56592	30.3477	27.9188
S1 3.2AO6	4.28503	11.2207	1.68479	12.5704	9.1674	3.31706	1.84405	0.36258
S10 1.EO2	20.5585	45.9309	9.55428	38.8368	29.6245	6.90352	7.79768	7.1066
S10 1.G03	5.63312	15.9679	3.99872	29.0807	19.9171	3.94006	2.31823	1.95794
S10 2.CO3	1.01107	13.9334	4.55321	9.94371	4.89765	11.8707	1.00105	1.74039
S10 2.GO1	3.89986	23.2429	7.69887	25.7036	22.5166	9.41236	9.32561	6.88905
S2 2.CO4	9.9663	14.0567	1.93005	83.8649	9.19252	4.32733	31.7176	65.6998
S2 2.EO3	38.8541	46.5783	5.68351	43.3396	3.30278	6.02795	100	1.45033
S2 2FO4	9.43669	11.4982	2.14331	9.38086	6.71857	2.89611	17.3867	2.6831
S3 2.AO4	15.7439	10.0185	1.68479	10.6942	7.44694	2.30679	2.73973	1.08774
S3 2.CO2	100	84.7102	6.09938	60.9756	19.7287	1.26284	10.8535	3.04569
S3 2.FO1	12.181	17.8792	1.15163	11.257	2.39859	0.33676	1.94942	0.58013
S3 2.FO4	8.6182	9.4328	0.98102	57.0356	7.03253	2.27311	20.8641	57.578
S3 2.FO6	2.11844	5.57953	1.43954	7.87992	4.54603	1.34703	0.79031	1.01523
S3 2.GO2	26.1435	12.762	1.59949	7.87992	1.56976	0.05051	4.21496	2.53807
S4 2.BO3	8.57005	18.0333	4.12668	23.0769	17.2422	6.14582	18.2824	4.27846
S4 2.BO4	11.3625	12.7004	0.82107	11.8199	5.13626	2.40781	7.63962	2.6831
S4 2.CO1	53.7795	49.9692	8.04009	42.7767	15.0823	2.08789	17.8609	4.78608
S4 2.CO3	1.73327	13.0395	2.00469	11.0694	6.1409	1.68379	1.00105	0.65265
S4 2.CO4	10.8329	39.6732	7.95479	34.7092	28.1678	8.11584	2.37092	3.84336
S4 2.EO5	17.6216	16.1837	3.40158	16.1351	9.48135	6.23001	6.00632	1.81291
S6 1.2BO4	14.5402	12.9162	6.0354	52.1576	26.9496	7.44233	33.7197	4.20595
S6 2.AO1	47.2316	24.0136	1.55684	30.5816	9.29298	3.50227	18.177	1.23278
S6 2.DO5	4.91093	4.68557	0.76775	8.25516	4.42044	0.94292	16.4384	0.94271
S6 2.EO4	3.08137	7.3058	0.9277	25.3283	8.89112	1.33019	99.8419	27.0486
S6 2.GO4	4.28503	7.18249	3.8601	26.6417	15.6348	4.17579	4.58377	3.91588
S7 2.BO3	11.6033	100	100	79.1745	100	100	15.0685	30.0218
S7 2.DO2	18.103	69.5129	39.0168	52.1576	69.3457	60.3637	22.1286	100
S8 2.2EO3	43.1873	59.9877	22.4355	100	8.94135	24.1792	76.3962	20.7397
S8 2.EO2	2.5999	21.7633	6.41928	12.0075	15.346	6.0953	7.74499	8.41189
C1 1.2AO6	41.573	49.6763	56.1437	15.9341	12.4146	56.2426	43.6546	0.98093
C1 1.2BO5	14.6067	15.4797	39.1304	6.73077	40.3189	16.0523	16.0611	32.4251
C1 1.2FO4	9.39734	6.8864	17.7694	21.2912	38.3827	14.3876	13.7967	32.6431
C1 1.2GO4	14.096	24.9559	50.2836	4.12088	17.0843	13.7931	19.5366	54.2234
C1 1.3AO2	31.5628	53.2078	14.1777	9.47802	13.6674	22.9489	6.26646	3.43324
C1 1.3BO2	7.55873	6.94526	10.586	3.15934	29.6128	4.87515	7.31964	18.4196
C1 1.3CO4	6.94586	2.5309	3.21361	1.92308	23.4624	1.30797	3.21222	0.32698

C1 1.FO5	100	97.2337	100	43.8187	56.3781	78.9536	100	0.54496
C1 2.2CO4	4.59653	14.538	1.51229	2.06044	6.60592	2.25922	1.36914	1.03542
C1 2.2FO3	2.24719	32.0188	5.48204	2.74725	14.123	1.66468	0.52659	0.54496
C1 2.CO6	26.2513	41.7304	5.10397	4.53297	20.615	3.9239	4.73934	1.08992
C1 2.DO5	23.5955	33.1371	2.45747	2.74725	13.5535	2.14031	4.73934	0.76294
C1 2.GO4	1.73647	2.17775	2.0794	0.13736	1.13895	0.35672	0.31596	0.21798
C3 1.GO6	5.2094	2.47204	1.32325	4.67033	19.1344	6.77765	0.42127	0
C4 2.2DO1	8.27375	7.76928	4.7259	6.31868	12.9841	5.11296	4.94997	6.32153
C5 1.CO5	11.0317	34.3732	97.7316	63.4615	100	100	26.6456	42.5613
C5 1.FO1	1.43003	1.35374	3.40265	0.82418	5.80866	1.66468	0.47393	0.43597
C5 1.GO2	0.91931	0	2.83554	0.68681	3.18907	1.78359	0.47393	0.38147
C5 1.GO5	0.91931	0.41201	2.0794	0.54945	2.2779	1.42687	0.10532	0.0545
C6 1.CO1	1.73647	2.11889	5.48204	4.94505	46.3554	4.75624	0.78989	0.92643
C6 1.GO4	1.83861	0.88287	0.56711	1.51099	10.2506	1.30797	0.42127	0.16349
C6 2.2CO6	28.8049	37.1395	44.8015	75.2747	8.76993	45.1843	54.1864	0.32698
C6 2.CO1	4.39224	2.47204	4.7259	6.31868	42.8246	7.13436	1.73776	1.08992
C6 2.DO1	15.4239	32.0188	36.1059	100	77.1071	62.1879	64.0864	100
C6 2.DO2	4.18795	3.29606	5.48204	8.1044	49.4305	6.06421	1.36914	1.47139
C7 2.DO2	0	0.76516	1.13422	0	3.53075	0.35672	0	0.10899
C7 3.AO5	28.907	45.7916	25.7089	37.7747	13.2118	19.3817	18.0621	23.8147
C7 3.CO2	37.3851	61.9776	62.949	29.9451	68.1093	39.8335	11.1111	43.3243
C8 1.2GO2	2.14505	6.82755	18.7146	3.57143	38.3827	6.42093	0.2633	0.38147
C8 2.FO2	59.7549	100	55.5766	75.8242	87.5854	68.1332	82.991	93.951

H3N2 rHA-specific antibodies

Antibody	A/Wisconsin/67/05	A/Uruguay/716/07	A/Hong Kong/1/68	A/Panama/2007/99
S2 2.BO4	3.09563	3.27186	5.7903	27.0408
S2 3.8CO3	19.7347	38.9054	0.15649	7.14286
S2 3.9CO5	9.56329	8.26889	0.93897	8.67347
S2 3.DO2	21.7247	40.8685	2.66041	82.1429
S3 2.GO6	23.8806	23.26	66.5102	73.4694
S3 3.BO6	40.5196	49.7323	100	100
S4 2.AO5	6.02543	2.32005	1.09546	19.898
S4 2.DO1	21.1719	7.1981	2.03443	38.7755
S4 2.EO2	42.7861	28.7329	24.2567	85.7143
S4 2.EO4	6.19127	3.39084	2.19092	87.7551
S5 3.5CO1	13.7645	23.9738	1.09546	10.7143
S6 1.BO1	15.9204	4.52112	10.0156	37.7551
S6 3.3EO6	22.6092	44.5568	1.09546	45.9184
S7 2.EO2	11.1111	2.85544	12.2066	28.5714
S8 1.GO5	8.56827	4.28316	11.2676	24.4898
S8 2.BO3	58.3748	43.843	51.4867	92.3469

S9 3.2BO3	100	100	5.32081	30.102
S9 3.3CO1	4.69873	6.78168	11.1111	43.8776
C1 1.2AO2	80.3993	51.9953	100	84.0909
C1 1.3BO1	9.2559	2.23005	9.39335	2.27273
C1 1.3EO3	5.80762	3.40376	5.28376	1.62338
C1 1.BO1	14.3376	10.2113	10.5675	13.961
C1 1.CO2	45.5535	38.8498	1.76125	100
C1 1.DO5	17.2414	7.39437	10.1761	8.11688
C1 1.GO1	7.6225	3.75587	10.3718	2.5974
C1 2.2FO4	84.9365	100	12.7202	35.7143
C1 2.EO5	23.2305	22.4178	51.272	12.6623
C1 3.7CO2	10.7078	1.52582	0.78278	2.5974
C1 3.7FO2	19.9637	10.2113	38.1605	56.8182
C2 2.2GO5	29.5826	16.6667	31.3112	46.4286
C2 3.FO4	16.8784	34.8592	0.19569	30.1948
C3 2.3CO1	4.17423	1.64319	0.97847	2.5974
C3 2.3FO2	100	2.58216	0.19569	18.1818
C6 1.CO4	3.81125	0.70423	0.78278	3.24675
C6 2.2EO5	13.2486	7.74648	2.93542	5.51948
C6 2.BO1	4.35572	1.29108	0.39139	1.94805
C6 3.BO2	4.35572	5.75117	1.17417	7.79221
C8 1.2AO2	15.245	8.4507	6.45793	13.961
C8 1.2CO3	18.6933	5.16432	2.73973	9.74026
C8 1.2EO6	12.5227	4.57746	4.10959	6.16883
C8 1.BO2	30.3085	5.28169	3.5225	10.3896

* Values shown here were used to plot S3 Fig. The values were normalized on a scale of 0-100% against an internal control for each strain to obtain relative level of binding of each antibody.
